# Current Advances on Structure-Function Relationships of Pyridoxal 5′-Phosphate-Dependent Enzymes

**DOI:** 10.3389/fmolb.2019.00004

**Published:** 2019-03-05

**Authors:** Jing Liang, Qian Han, Yang Tan, Haizhen Ding, Jianyong Li

**Affiliations:** ^1^Department of Biochemistry, Virginia Polytechnic Institute and State University, Blacksburg, VA, United States; ^2^Laboratory of Tropical Veterinary Medicine and Vector Biology, Hainan Key Laboratory of Sustainable Utilization of Tropical Bioresources, Institute of Agriculture and Forestry, Hainan University, Haikou, China; ^3^Institute of Synthetic Biology, Shenzhen Institutes of Advanced Technology, Chinese Academy of Sciences, Shenzhen, China

**Keywords:** pyridoxal 5′-phosphate, structure-function relationship, reaction mechanism, amino acid residues, reaction specificity

## Abstract

Pyridoxal 5′-phosphate (PLP) functions as a coenzyme in many enzymatic processes, including decarboxylation, deamination, transamination, racemization, and others. Enzymes, requiring PLP, are commonly termed PLP-dependent enzymes, and they are widely involved in crucial cellular metabolic pathways in most of (if not all) living organisms. The chemical mechanisms for PLP-mediated reactions have been well elaborated and accepted with an emphasis on the pure chemical steps, but how the chemical steps are processed by enzymes, especially by functions of active site residues, are not fully elucidated. Furthermore, the specific mechanism of an enzyme in relation to the one for a similar class of enzymes seems scarcely described or discussed. This discussion aims to link the specific mechanism described for the individual enzyme to the same types of enzymes from different species with aminotransferases, decarboxylases, racemase, aldolase, cystathionine β-synthase, aromatic phenylacetaldehyde synthase, et al. as models. The structural factors that contribute to the reaction mechanisms, particularly active site residues critical for dictating the reaction specificity, are summarized in this review.

## Introduction

Pyridoxal 5′-phosphate (PLP) is one of the active forms of vitamin B_6_, which is produced by pyridoxal kinase-mediated reactions. PLP-dependent enzymes catalyze a wide variety of reaction types and usually have a conserved lysine residue in the active site for PLP binding. The ε-amino group of the lysine residue and the aldehyde group of PLP forms a Schiff-base structure. Because this Schiff-base structure is linked through a protein-associated lysine residue, it is commonly referred as internal aldimine. After substrate (amino acid or amine) binding, the internal aldimine breaks up and a new Schiff base structure is formed between the amino group of substrate and aldehyde group of PLP via a *gem*-diamine intermediate (Fukui and Soda, 2008). This newly formed Schiff base is generally termed external aldimine to distinguish it from Schiff-base structure linked with the lysine residue in proteins. The aldimine exchange has been termed transaldimination. This external aldimine formation is common in many PLP-containing enzymes, but once external aldimine is formed, the subsequent reaction mediated by any given enzyme differs, which is dictated primarily by active site conformation or more specifically by the biochemical characteristics of the active site residues that interact with the specific chemical groups of the external aldimine.

In addition to the diversity of reaction specificity, PLP-dependent enzymes are involved in many key cellular processes and metabolism. These enzymes are involved in the amino acid metabolism and production of amino acid-derived metabolites. For example, L-3,4-dihydroxyphenylalanine (L-dopa) decarboxylase (DDC) catalyzes the decarboxylation of L-dopa and 5-hydroxytryptophan to produce dopamine and serotonin, respectively. Both dopamine and serotonin are neurotransmitters for not only mammals but also many other species. The regulation problems and deficiency of PLP-dependent enzymes caused probably by pathogenic mutations, lead to several metabolism symptoms. For example, the deficiency of DDC leads to development delay, abnormal movement, and other neurotransmitter-related symptoms. Deficiency of alanine-glyoxylate aminotransferase is involved in the primary hyperoxaluria type I disease while ornithine aminotransferase deficiency contributes to vision problems at night or under dim light (Cellini et al., 2014). There is also an inverse association between the blood PLP level and the risk of colorectal cancer (Larsson et al., 2010). In addition, it was reported and evaluated recently, for the first time that the insufficient PLP or B_6_ intake from food might contribute to pancreatic islet autoimmunity and the development of type I diabetes (Rubí, 2012).

As a result of diverse reaction specificity and the physiological significance, PLP-dependent enzymes are the foci of enzyme structure-function relationship studies. With the technological and methodological development, especially the sequencing of more genomes, technical progress of protein expression and purification, and the determination of more enzyme crystal structures, a better understanding of enzymatic catalysis is achieved, especially how the active site residues facilitate PLP to increase the specific reaction and at the same time decrease the possibility of side reactions. In this review, we focus on mechanisms involved in initial catalytic steps shared by most PLP-dependent enzymes and try to link the mechanism of one enzyme to the reaction specificity mechanism of enzymes with similar reaction specificity from different species.

## New Insights Into PLP Chemistry in Catalysis

PLP acts as a cofactor in these enzymes because of its specific properties. PLP has one heteroaromatic pyridine ring, one aldehyde group, a hydroxyl group, and a phosphate group.

The aldehyde group of PLP makes it possible to form imine with free amino group (e.g., the internal aldimine, formed between PLP and the conserved lysine residue and external aldimine formed between PLP and substrate amino group). PLP binding in the active site through phosphate group in some exceptions are also observed (Eliot and Kirsch, 2004). Another unprotonated primary amino group can attack the internal aldimine or external aldimine formed to make the internal or external aldimine reversible. The ability to form reversible imine allows PLP binding, substrate binding, product release and regeneration of enzyme with PLP bond in the active site.

The heteroaromatic pyridine ring of PLP enables it to stabilize carbanionic intermediate formed for most (if not all) of the PLP-dependent enzymes, except aminomutase family (radical-initiated reaction) (Frey, 2001). The electrons of the carbanionic intermediate are resonance stabilized and delocalized by the electron-sink of PLP. Quinonoid intermediates, proposed as the key intermediates in many PLP-dependent catalytic mechanisms, are the resonance forms of carbanionic intermediates, but quinonoid intermediates have not been actually observed in some reactions, such as, the reaction catalyzed by alanine racemase. In alanine racemase, the unprotonated pyridine nitrogen made it difficult to form a quinonoid intermediate, which might be destabilized and could be a transition state in the reaction catalyzed by alanine racemase (Major and Gao, 2006).

The functions of other groups of PLP were also discussed. The hydroxyl group could function as a proton donor or acceptor, which will be discussed in detail in the mechanism part. The role of 5′-phosphate group was less mentioned or elaborated. It was proposed that the phosphate group of PLP played a role as a general acid/base for accepting or donating a proton in the reaction catalyzed by glycogen phosphorylase (Helmreich, 1992; Livanova et al., 2002). The phosphate group of PLP is in close proximity to the substrate phosphates (Parrish et al., 1977; Chang et al., 1983; Livanova et al., 2002). In the forward reaction, the glycoside oxygen is protonated by orthophosphate and PLP becomes a dianion by donating one proton to the substrate phosphate. Then a covalent ligation of substrate phosphate with the intermediate leads to glucose 1-phosphate formation and PLP is converted back to a monoanion (Helmreich, 1992; Livanova et al., 2002). The importance of the phosphate group in PLP was also studied recently through comparative analysis using pyridoxal and pyridoxal 5′-phosphate as the cofactor in serine palmitoyltransferase. Although pyridoxal could associate with a conserved active site lysine residue and transaldimination reactions proceeded, a replacement of PLP with pyridoxal lowered more than 10-fold of its enzyme activity (Beattie et al., 2013). The phosphate group of PLP was proposed to interact with the substrate L-serine hydroxyl group and contributed to the critical intermediate formation and stereospecific orientation of formed quinonoid or carbanionic intermediates (Beattie et al., 2013). The phosphate group was also suggested functioning as an acid/base catalyst to promote proton transfer to aid in external aldimine formation and accelerating *gem-diol* intermediate formation in kynureninase-mediated hydrolytic cleavage reaction (Phillips et al., 2014).

**Figure 1:**
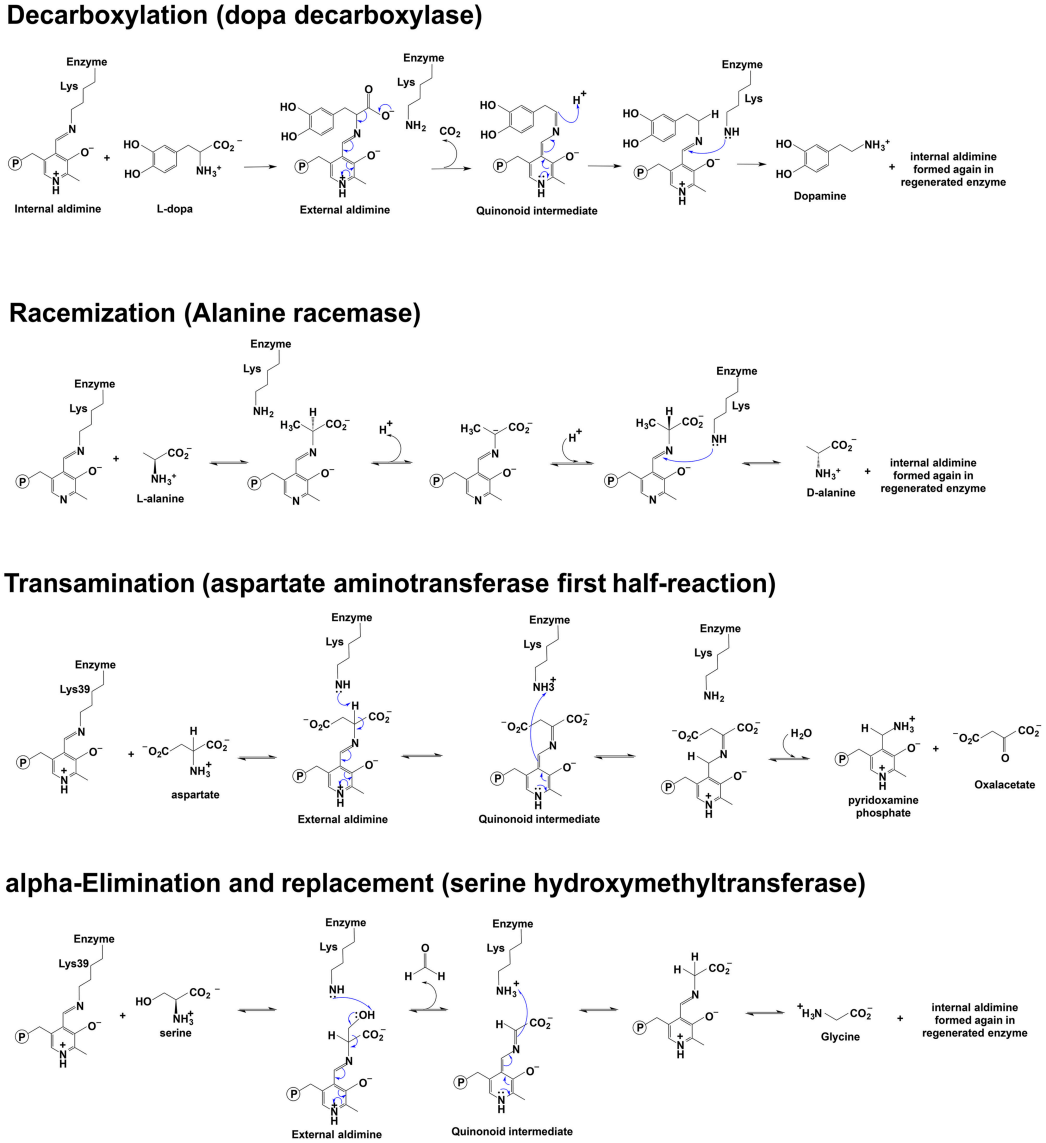
Scheme depicting of examples of the reaction mechanisms catalyzed by PLP-dependent enzymes. Reaction mechanisms of decarboxylation, racemization, transamination, and α-elimination and replacement are shown (Watanabe et al., 1999, 2002; Eliot and Kirsch, 2004; Toney, 2014).

Because of the chemical role of PLP and different interactions with the enzymatic environment (e.g., active site residues), the reaction direction of PLP-dependent enzymes differs (e.g., decarboxylation, racemization, transamination, elimination, replacement, et al.) (Figure 1). Approximately 4% of all classified enzyme activities are PLP-dependent (Clausen et al., 1996; Percudani and Peracchi, 2009). Racemization is through deprotonation on one side and reprotonation of Cα on the opposite side, while a reprotonation at C4′ of PLP following deprotonation at Cα is a critical step of transamination for a ketimine intermediate formation. The α, β-elimination is dependent on the leaving group at the β position. Decarboxylation is through the removal of –COO^−^ group from the external aldimine to form a carbanionic or quinonoid intermediate and followed by protonation of the intermediate at Cα to form an amine. Retro-aldol condensation is through bond breaking between Cα and Cβ to form a carbanionic intermediate (Toney, 2011). Some enzymes catalyze a combination of different reaction types, e.g., dialkylglycine decarboxylase (DGD), which catalyzes the decarboxylation-dependent transamination (Fogle and Toney, 2010; Taylor et al., 2015).

## Fold Types of PLP-Dependent Enzymes

Grishin et al. classified PLP-dependent enzymes into 5-fold types and pointed out that some enzyme families could not be categorized into these 5-fold types and might be arranged to additional fold types (Grishin et al., 1995). Subsequently, PLP-containing proteins were separated into seven clusters (Percudani and Peracchi, 2009), which contained the 5-fold types suggested by Grishin et al. (1995) and two additional clusters assigned as fold types VI (including D-lysine-5,6-aminomutase) and VII (containing lysine 2,3-aminomutase) (Percudani and Peracchi, 2009).

Fold type I includes aminotransferases (except aminotransferase class IV), decarboxylase groups II and III (Grishin et al., 1995; Steffen-Munsberg et al., 2015) and some enzymes with α-, β- or γ-elimination activity (Kappes et al., 2011). These fold type I enzymes usually have their Schiff base Lys residue near C-terminus, a glycine-rich loop (involved in binding of PLP phosphate group) and a hydrophobic β-strand before the Lys residue. This fold group usually contains a conserved aspartate residue that interacts with the PLP ring N atom and this residue is 20–50 amino acids preceding the Lys residue (Grishin et al., 1995). Fold type I enzymes usually exist as homodimers or homotetramers and each subunit contains a PLP molecule, but their active site is located at the interface between subunits and is composed of residues from two subunits (most residues are from one subunit) at the interface (Schneider et al., 2000; Han et al., 2010b; Milano et al., 2013). Although the dimer is typically the minimum assembly required for catalytic activity, the active site of *Escherichia coli* L-threonine aldolase tetramer containing residues from three monomers was also reported (Di Salvo et al., 2014). The subunit of fold type I enzymes contains a large domain and a small domain. The large domain consists of a seven-stranded β-sheet at the N-terminal. The small domain (the C-terminal of enzymes) folds into a 3- or 4-stranded β-sheet covered with helices on one side (Schneider et al., 2000; Han et al., 2010b). Structural alignment further classified the fold type I enzymes into six subclasses according to the structure of the N-terminal part (Mehta et al., 1993; Grishin et al., 1995; Käck et al., 1999; Schneider et al., 2000).

Tryptophan synthase β-family is the representative enzyme of fold type II. Other enzymes (e.g., cysteine synthase, serine dehydratase, threonine dehydratase, *O*-acetyl serine sulfhydrylase, threonine synthase, etc.) also belong to fold type II (Grishin et al., 1995). Fold type II enzymes, in contrast with fold type I enzymes, usually have the Schiff base Lys residue closer to N-terminus, while the loop region for PLP phosphate group binding is near the C-terminus. There are two β-strands flanked by α-helices in fold type II enzymes. Glu350 in tryptophan synthase is involved in PLP binding and Asp or Ser are also conserved at the equivalent position suggested by sequence alignment of fold type II enzymes (Percudani and Peracchi, 2009). Different from fold type I to have one Asp residue to interact with the pyridine nitrogen, a serine residue is conserved in fold type II enzymes at the equivalent position (Schneider et al., 2000). Fold type II enzymes are functional as dimers, tetramers, or oligomers with the active site being composed of residues all from one subunit (Milano et al., 2013) and many fold type II enzymes are under allosteric control. Tryptophan synthase exists as an α_2_β_2_ tetramer with α and β being the regulatory subunit and the catalytic subunit, respectively. Threonine deaminase is a homodimer and has one catalytic domain and one regulatory domain in each subunit. The region for PLP-binding in fold type II enzymes contains an N-terminal domain and a C-terminal domain with similar size to each other (Schneider et al., 2000).

Fold type III enzymes (e.g., alanine racemase and eukaryotic ornithine decarboxylase) have similar arrangement for aldimine-forming Lys residue (closer to N-terminus) and PLP interacting glycine-rich loop (near C-terminus) as those of fold type II enzymes, but fold type III enzymes have a hydrophobic β-strand before the conserved Lys residue and two β-strands in the region of β/α units (Grishin et al., 1995). Alanine racemase and human ornithine decarboxylase are active as dimers. Alanine racemase consists of two domains in each subunit. One domain is composed of eight-stranded α/β barrel and another one contains β strands. In alanine racemase, PLP binding site is in a cleft between these two domains and one arginine residue interacts with the pyridine ring nitrogen atom through forming a hydrogen bond (Schneider et al., 2000). In mammalian ornithine decarboxylases, a glutamic acid residue is at the equivalent position interacting with the pyridine ring nitrogen atom (Kern et al., 1999).

D-alanine aminotransferase family and branched chain aminotransferase were assigned as fold type IV enzymes (Grishin et al., 1995; Okada et al., 1997; Percudani and Peracchi, 2009; Kappes et al., 2011). The fold type IV enzymes are usually functional as homodimers, and branched-chain aminotransferase is a hexamer. There are two domains. The N-terminal domain includes a six-stranded antiparallel β sheet and two α helices. The C-terminal domain consists of a pseudo-β-barrel and some helices. A glutamate acid residue interacts with the pyridine ring nitrogen atom (Schneider et al., 2000).

The glycogen phosphorylase family was classified as fold type V enzyme, which contains a PLP-binding domain with a lactate dehydrogenase fold (Grishin et al., 1995). For fold type V enzymes, PLP phosphate group is involved in proton transfer in the enzymatic reaction (Schneider et al., 2000). Different from other fold types listed above, glycogen phosphorylase has no hydrogen bond interactions between the active site residues and the pyridine ring nitrogen atom of PLP (Schneider et al., 2000). Glycogen phosphorylase has two forms (phosphorylase *a* and *b*) and exists as tetramer or dimer. The conversion from phosphorylase *b* to *a* is through phosphorylation of phosphorylase protomer *b* catalyzed by phosphorylase *b* kinase while the reverse reaction is mediated by phosphorylase phosphatase (Cohen et al., 1971). A study on phosphorylase *a* indicated that both dimer and tetramer enzymes could bind glycogen, but the tetramer bound the substrate with lower affinity and had no activity. The glycogen binding lowers the association rate of the tetramer formation from dimer, but the dissociation of tetramer to form dimer is not affected by glycogen binding. The existence of both dimer and tetramer form of glycogen phosphorylase might be involved in a regulation mechanism for the glycogen metabolism (Wang, 1999). There are three domains in glycogen phosphorylase, including an N-terminal domain, a glycogen-binding domain, and a C-terminal domain (Schneider et al., 2000).

Lysine 5,6-aminomutase family and the lysine 2,3-aminomutase family were labeled as fold type VI and VII, respectively (Percudani and Peracchi, 2009). Lysine 5,6-aminomutase is an α_2_β_2_ tetramer. The α subunit is larger and contains a triosephosphate isomerase barrel domain and several α helices and β-strands. The smaller β subunit includes an N-terminal domain and the Rossmann domain for adenosylcobalamin or coenzyme B12 binding and PLP binding (Berkovitch et al., 2004). The lysine-2,3-aminomutase was crystallized as a homotetramer, which is a dimer of domain-swapped dimers (Lepore et al., 2005). Other aggregation states (e.g., dimer, hexamers) of the lysine-2,3-aminomutase are also suggested by cross-linking studies (Cho et al., 1997). Each subunit has three domains, including a central globular domain, an N-terminal domain, and a C-terminal domain. Different from lysine-5,6-aminomutase, lysine-2,3-aminomutase is dependent on PLP, S-adenosyl-L-methionine, and an iron-sulfur [4Fe-4S] cluster as cofactors. The cofactor-binding site in the subunit is a channel formed by six β/α (Lepore et al., 2005).

The enzymes reviewed in this study belong to fold type I to IV. Enzymes catalyzing a reaction type include enzymes from different fold types and a fold type is composed of enzymes catalyzing diverse kinds of reactions. For example, aminotransferases belong to the fold type I and IV, and alanine racemase and serine racemase belong to fold type III and II, respectively (Watanabe et al., 2002; Canu et al., 2014). Both aromatic amino acid decarboxylase and fruit fly phenylacetaldehyde synthase are fold type I enzymes. This review focuses on residues involved in reaction specificity of structurally and mechanistically related enzymes. Serine hydroxymethyltransferase, L-threonine aldolase, and fugal alanine racemase are fold type I enzymes and have similar active site residues and all have half-transamination, aldol cleavage, and racemization activity (Contestabile et al., 2001; Di Salvo et al., 2013, 2014). Aromatic amino acid decarboxylase and phenylacetaldehyde synthase may share similar reaction steps from external aldimine formation, decarboxylation, to the carbanionic intermediate formation, but deviate from each other by the different ability to protonate the carbanionic intermediate at Cα by varied active site residues (Liang et al., 2017). In addition to protonation efficiency differences, the deprotonation and protonation stereo-specificity are observed between racemase and aminotransferase. Racemases catalyze deprotonation at Cα of the substrate on one side and reprotonation on the another side at Cα while deprotonation at Cα of substrate and reprotonation at C4′ of PLP are the key steps in reactions catalyzed by aminotransferase (Eliot and Kirsch, 2004; Toney, 2011). Cystathionine β-synthase is a member of fold type II (Miles and Kraus, 2004) and catalyzes β-replacement reactions. Similar to reactions catalyzed by racemase and aminotransferase, the first step of reactions catalyzed by cystathionine β-synthase is proton abstraction from Cα. These functionally and mechanistically related enzymes are reviewed here (Koutmos et al., 2010).

## Mechanism of Reaction Specificity

### Stereoelectronic Effects: Unifying Enzymatic Mechanism

The structural characteristics of PLP make it possible to undergo a number of reactions. Its pyridine ring could function as an electron sink for delocalization and contribute to the resonance stabilization of the electrons or negative charges developed from bond breaking at Cα. However, in a given buffer, mixing of PLP and amino acid or amine does not lead to detectable specific reactions as compared with reactions catalyzed by PLP-dependent enzymes (the rate of reaction is too slow to be physiologically relevant). Only the combination of given protein component and PLP makes the PLP-dependent enzymes work efficiently on the specific substrate and specific reaction direction. One might ask how a given enzyme controls the reaction specificity because PLP functions in most PLP-dependent reactions to delocalize and stabilize the electrons or negative charge (developed from bond breaking at Cα) in the transition state.

In 1966, Dunathan proposed a unified theory explaining the reaction specificity of PLP-dependent enzymes (Dunathan, 1966). It was hypothesized that, if the delocalization energy was gained after the loss of one group from Cα, the PLP-dependent enzyme should bind the substrates in a specific geometry to have the bond to be broken in a perpendicular plane to the plane defined by pyridoxal imine (the π system) to make the bond labile to be broken (Dunathan, 1966). For example, the α-decarboxylase was predicted to bind the substrates in a specific orientation to have the -COO^−^ group perpendicular to the plane of Schiff base and PLP ring. This orientation was proposed to facilitate decarboxylation process.

### Enzymatic Mechanism

#### External Aldimine Formation

After substrate binding, the incoming substrate interacts with the internal aldimine through forming an unstable *gem*-diamine intermediate, and that leads to breaking up of internal aldimine and formation of external aldimine. This process is commonly termed transaldimination.

It has been generally considered that a nucleophilic attack of the substrate amino group at C-4' of the internal aldimine was the first step during transaldimination. To promote this reaction, it was proposed that the aldimine nitrogen should be protonated and this topic has been discussed in detail (Spies and Toney, 2003; Chan-Huot et al., 2013). In addition to the aldimine nitrogen, some recent structural analyses of a PLP-containing tyrosine phenol-lyase suggested that the interactions of aspartate residue and pyridine ring N atom might play a role in favoring transaldimination. The site-directed mutagenesis of Asp214 to Ala led to deprotonation of the pyridine ring N, the reduced reaction activity, one assumed relaxed geometry and the increased stability of the internal aldimine. It was proposed that this observed stabilization of the ground state in D214A variant might reduce the rate of the external aldimine formation, while Asp214 in wild-type enzyme might maintain the strained state of the internal aldimine, which likely led to the acceleration of transaldimination (Milić et al., 2012). The Asp residue is usually conserved, but in some enzymes, this residue is replaced by a Glu, which can interact with PLP pyridine ring N in the same manner (forming salt bridge) in fold type I and IV enzymes. In fold type II enzymes, the PLP pyridine ring N also interacts with Ser or Thr or Cys through forming hydrogen bonds. Whether these residues also have a role in different fold type enzymes as the Asp does in favoring transaldimination needs further elucidation.

To proceed nucleophilic attack on PLP C4′ atom, the substrate amino group must be a neutral group (not positively charged) in order to function as a nucleophile. In a reaction mixture under the physiological condition (pH around 7), the substrate amino group is usually protonated according to the pKa of the amino group. This leads to another essential question as to how the amino group of a substrate is deprotonated. A conformational study of one PLP-dependent enzyme suggested that nucleophilic attack of the substrate was achieved by a molecular switch of the dihedral angle between pyridine ring and Schiff-base linkage. PLP O3′ functions as a proton acceptor to make the nucleophile available (Ngo et al., 2014). Whether this is the typical way or mechanism for deprotonation of substrate amino group in other PLP-dependent enzymes needs to be analyzed.

#### Aminotransferase

Aminotransferase catalyzes the reversible transformation between an amino acid and α-keto acid. After external aldimine formation, deprotonation at Cα leads to a carbanionic intermediate or quinonoid intermediate. Reprotonation at C4′ position of PLP leads to a ketimine intermediate and one H_2_O molecule is added to the intermediate Cα. The half-reaction proceeds to have α-keto acid involved to regenerate PLP and another amino acid. This is a commonly accepted reaction mechanism. In the process, proton transfer between Cα and C4′ was suggested to be promoted by the conserved Lys residue, which is involved in the formation of internal aldimine. The role Lys residue plays in proton transfer was supported by a greatly decreased rate of catalysis in enzyme variant (Toney and Kirsch, 1993). In literature, there has been a debating issue on the concerted or stepwise mechanism of proton transfer between Cα and C4′ (Goldberg and Kirsch, 1996; Toney, 2014). The existence of quinonoid intermediate was indicated by light enhancement of aspartate aminotransferase-catalyzed reaction and also by low concentrations of quinonoid intermediate in steady state (Toney, 2014).

**Figure 2:**
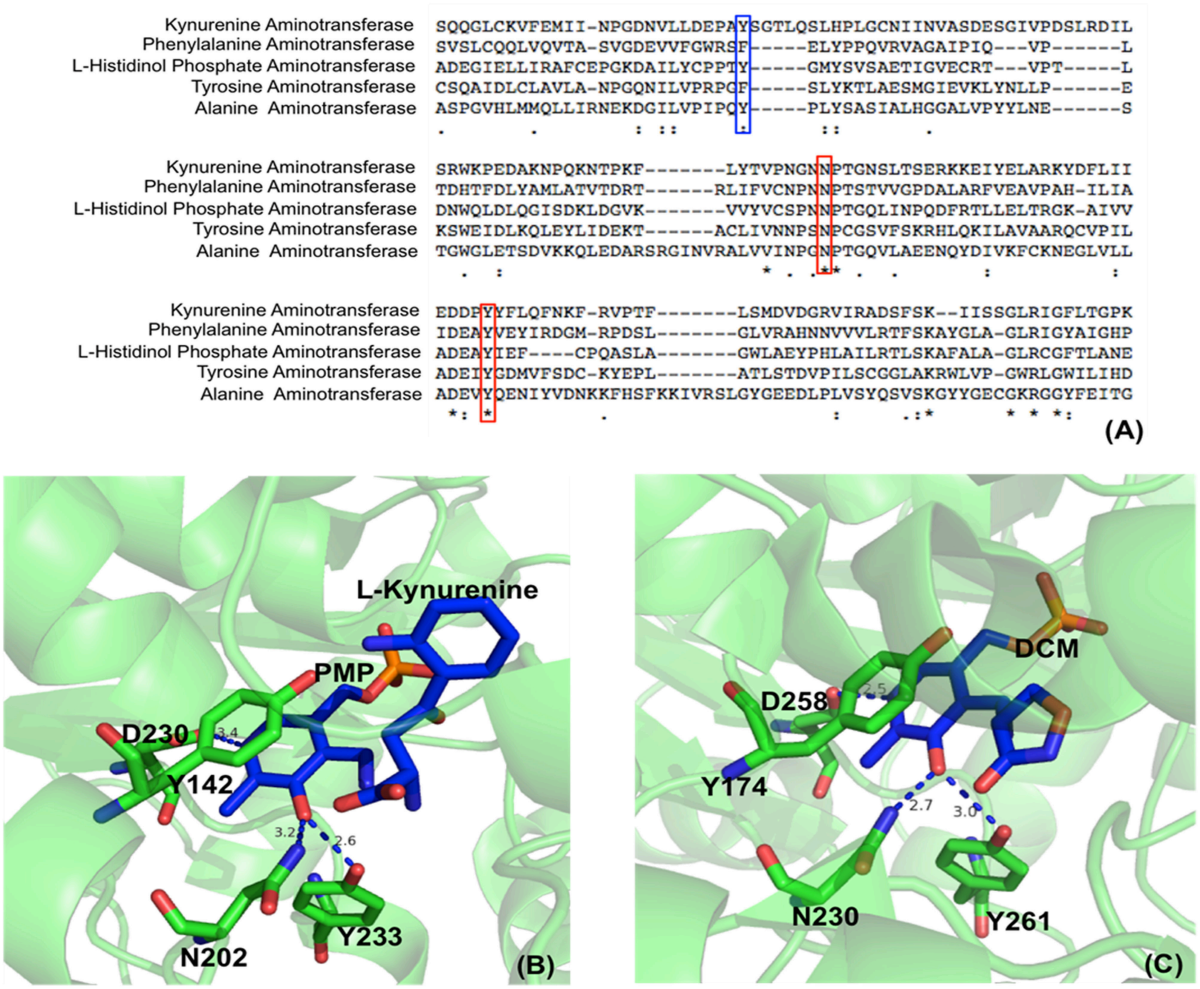
Partial protein sequence alignment of aminotransferases, and the position of three conserved residues in the active site. Sequences of *Homo sapiens* kynurenine aminotransferase (Accession: NP_057312.1), *Mycobacterium tuberculosis H37Rv* phenylalanine aminotransferase (PDB: 4R2N, chain A) (Nasir et al., 2016), *Enterobacteriaceae* L-histidinol phosphate aminotransferase (Accession: WP_000108941.1), *Homo sapiens* tyrosine aminotransferase (PDB: 3DYD, chain A) (http://www.rcsb.org/structure/3DYD) and *Hordeum vulgare* alanine aminotransferase (PDB: 3TCM, chain A) (Duff et al., 2012) are shown. The uncharged aromatic amino acid residues for π-π stacking with PLP ring are highlighted in blue box while the Asn and Tyr residues forming hydrogen bonds with PLP O3′ are highlighted in red boxes **(A)**. The Asn and Tyr residues forming hydrogen bonds with O3′ of PLP are shown in green sticks while the ligands pyridoxamine phosphate (PMP), L-kynurenine and D-pyridoxyl-N,O-cycloserylamide-5-monophosphate (DCM) are shown in blue sticks. The uncharged aromatic amino acid residues forming π-π stacking with PLP pyridine ring and the Asp residue near pyridine ring N atom are also shown in green sticks. The distances between PLP O3′ and Asn or Tyr and the distances between pyridine ring N and Asp side chain are in blue dashed line and labeled (Unit: Å). L-kynurenine aminotransferase (PDB: 2R2N) (Han et al., 2008b) **(B)** and alanine aminotransferase (PDB: 3TCM) (Duff et al., 2012) **(C)** are presented as two examples.

Aminotransferases have an uncharged aromatic amino acid residue (Phe or Tyr or Trp, depending on different aminotransferases) for π-π stacking of the PLP ring (Figure 2). The presence of uncharged aromatic amino acid residue (Trp, Tyr, and Phe) for π-π stacking likely enhances the proton transfer opportunity between Cα and C4′ through Lys residue by leaving more lifetime for the intermediate to allow proton delivery by Lys residue.

**Figure 3:**
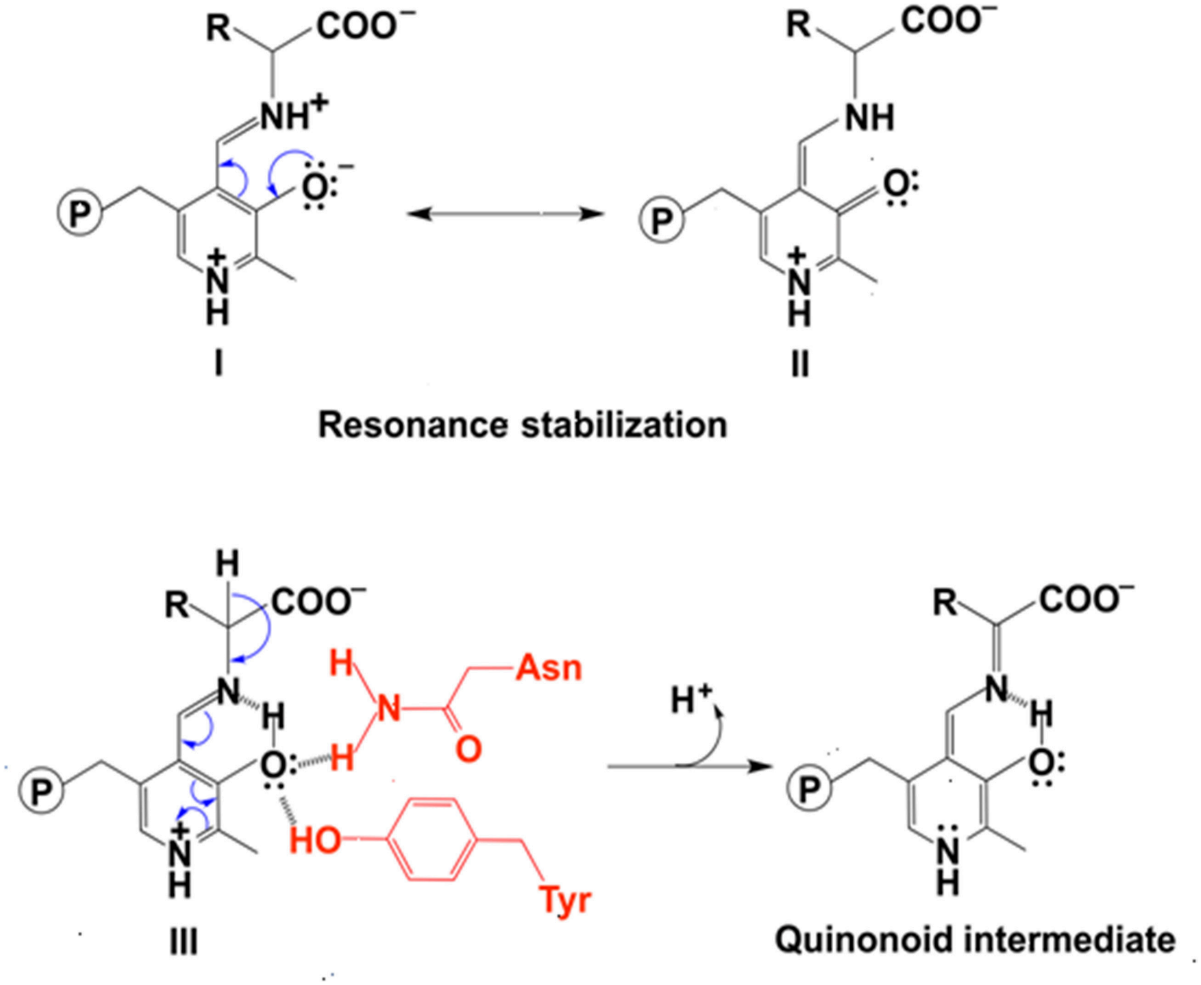
The effect of protonation states of O3′ on delocalization. The stabilization of ionized O3′ (form I) through resonance structure (form II). The electron withdrawing effect of PLP with a neutral O3′ (form III) and formation of quinonoid intermediate (Toney, 2014) are shown.

In addition to uncharged aromatic amino acid for π-π stacking with PLP, there are two active site residues Asn and Tyr that are conserved in aminotransferases (Figure 2) and form hydrogen bonds with PLP phenol group O3′ (Figure 2) (Sivaraman et al., 2001; Han et al., 2008a,b, 2010a,b; Duff et al., 2012; Nasir et al., 2016) (http://www.rcsb.org/structure/3DYD). By analyzing the catalytic mechanism, we propose that although ionized forms of phenol group could also possibly exist (Figure 3, form I and II), the neutral forms are likely predominant in the catalysis because ionized O3′ has more lone pairs of electrons (Figure 3, form I) than that of the neutral form (Figure 3, form III) and needs to be delocalized into the pyridine ring for stabilization (Figure 3, I and II). The delocalization effect of more lone pair electrons of ionized O3′ may counteract with the electron delocalization effect of reaction intermediates. These two residues likely help most of O3′ in neutral 3′-OH form. The neutral 3′-OH group, stabilized by hydrogen bonds with neutral Tyr and Asn, may better facilitate carbanionic intermediate formation and delocalization of electrons of the intermediate (Figure 3, form III) and thus may better promote deprotonation of Cα than other ionized form (Figure 3, form III and quinonoid intermediate). The conservation of these two residues for hydrogen bond formation and the predicted role in electron delocalization and intermediate stabilization suggest the importance of the interconverting intermediate stability. This is in agreement with the stepwise mechanism of aminotransferase.

Results derived from neutron crystallography of aspartate aminotransferase suggested that the O3′ was deprotonated in both internal aldimine and external aldimine (Dajnowicz et al., 2017). Whether, the O3′ is protonated or deprotonated after Cα deprotonation remains to be substantiated. Likewise, the possible role of O3′ protonation state in the carbanionic intermediate formation after Cα deprotonation needs further elucidation.

The roles of one flexible Arg residue that interacts with the carboxylate group of substrates in aminotransferases were also reported. ω-aminotransferase is active on multiple substrates, e.g., hydrophobic amines and amino acids. A density functional calculation study on *Chromobacterium violaceum* ω-aminotransferase suggested that the conformation (pointing its side chain toward or away from the active site) of Arg residue was a switch of recognition between the dual substrates (amino acids or hydrophobic amines). The reaction mechanisms of deamination for both substrates were similar, but reaction energetics was quite different. With the side chain of Arg416 positioned inside the active site, the half-transamination reaction from alanine to pyruvate was able to proceed because Arg416 aided in alanine binding through a salt bridge interaction with the carboxylate group of substrates. With the (S)-phenylethylamine as the substrate, the side chain of Arg416 pointed away from the active site (Manta et al., 2017). Similar roles of Arg residues were also reported in other aminotransferases. *Escherichia coli* aspartate aminotransferase has Arg292 and Arg386 to interact with the dicarboxylic substrates. When the side chain of Arg292 was pointing toward the active site, the enzyme had a higher binding affinity for dicarboxylic products and substrates. Arg386 interacts with the carboxylate group of aromatic substrates and dicarboxylic substrates (Chow et al., 2004). Arg414, which was the only arginine residue in the active site of *Paracoccus denitrificans* ω-aminotransferase, was found to recognize carboxylate group of substrates (Park et al., 2012). Arg417 was at the equivalent in a class III aminotransferase (PDB: 3HMU) from *Silicibacter pomeroyi* and was proposed to be involved in substrate promiscuity because of its flexibility (Rausch et al., 2012).

#### PLP-Dependent Aromatic Amino Acid Decarboxylase

In PLP-dependent decarboxylase, PLP serves as an electron sink to delocalize the unbounded electrons and to orientate enzyme-substrate in a specific orientation, both of which contribute to the decarboxylation and formation of a quinonoid intermediate. The protonation at Cα leads to an imine formation, which is attacked by Lys amino group to lead to one Schiff base formation between Lys residue and the PLP (internal aldimine). At the same time, the amine product is released. This is the generally accepted mechanism for the decarboxylation process.

**Figure 4:**
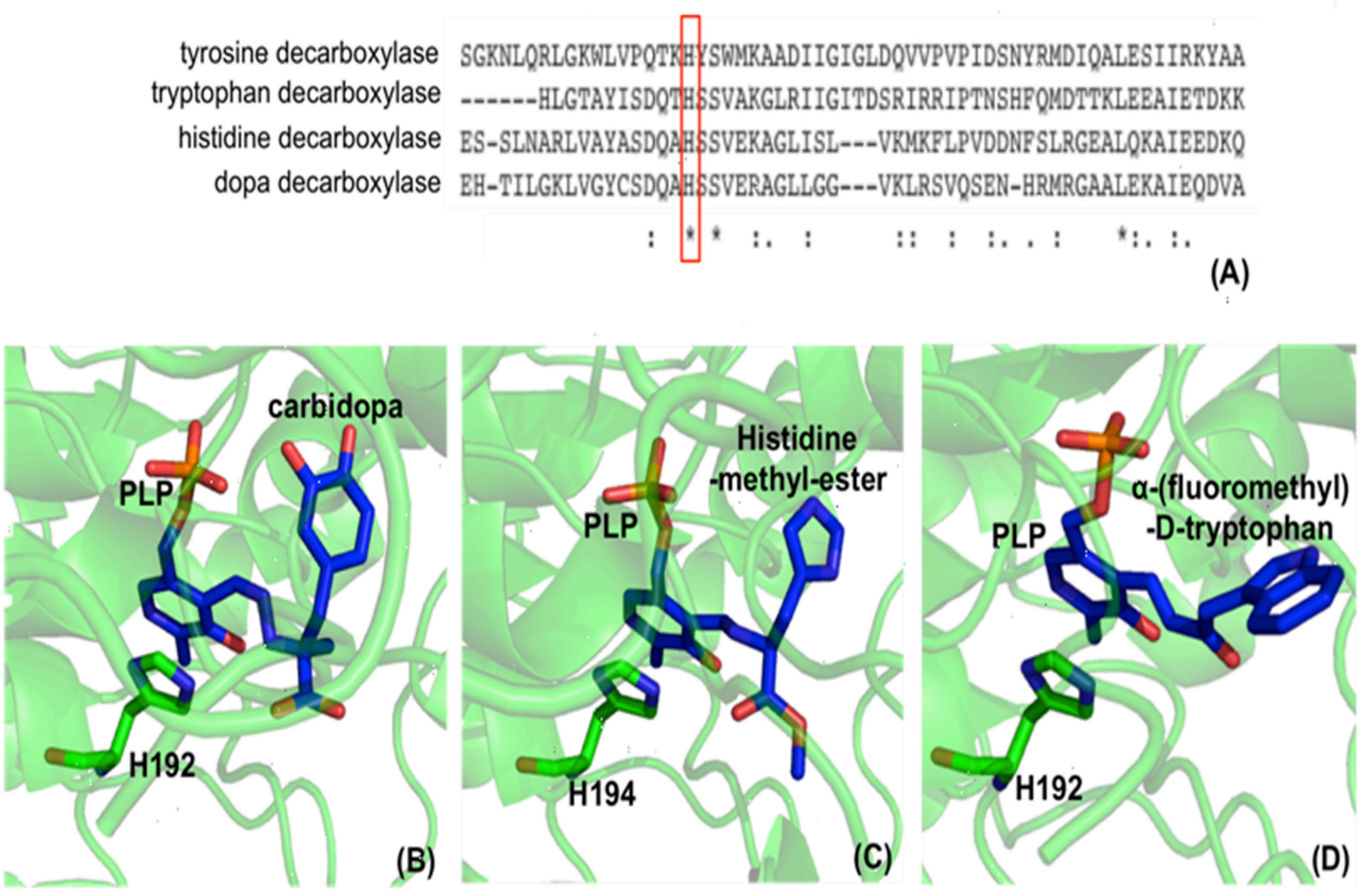
Partial protein sequence alignment of aromatic amino acid decarboxylases, and the positions of the conserved His residue in aromatic amino acid decarboxylases. The conservation of His192 residue (residue number is from *Drosophila melanogaster* dopa decarboxylase) in *Lactobacillus brevis* tyrosine decarboxylase (PDB: 5HSJ, chain A) (Zhu et al., 2016), *Ruminococcus Gnavus* tryptophan decarboxylase (PDB: 4OBU, chain E) (Williams et al., 2014), *Homo sapiens* histidine decarboxylase (4E1O_A) (Komori et al., 2012), and *Drosophila melanogaster* dopa decarboxylase (Accession: NP_724164.1) is shown and is highlighted in red box **(A)**. The dopa decarboxylase complexed with PLP and substrate-like inhibitor carbidopa [PDB: 1JS3 (Burkhard et al., 2001)] **(B)**, the histidine decarboxylase complexed with PLP and substrate analog histidine-methyl-ester [PDB: 4E1O (Komori et al., 2012)] **(C)**, and tryptophan decarboxylase complexed with PLP and substrate analog α-(fluoromethyl)-D-tryptophan [PDB: 4OBV (Williams et al., 2014)] **(D)** are shown. The external aldimines formed by PLP and substrate analogs are colored in blue and the conserved His residues in each decarboxylase critical for decarboxylation activity are shown in green sticks.

**Figure 5:**
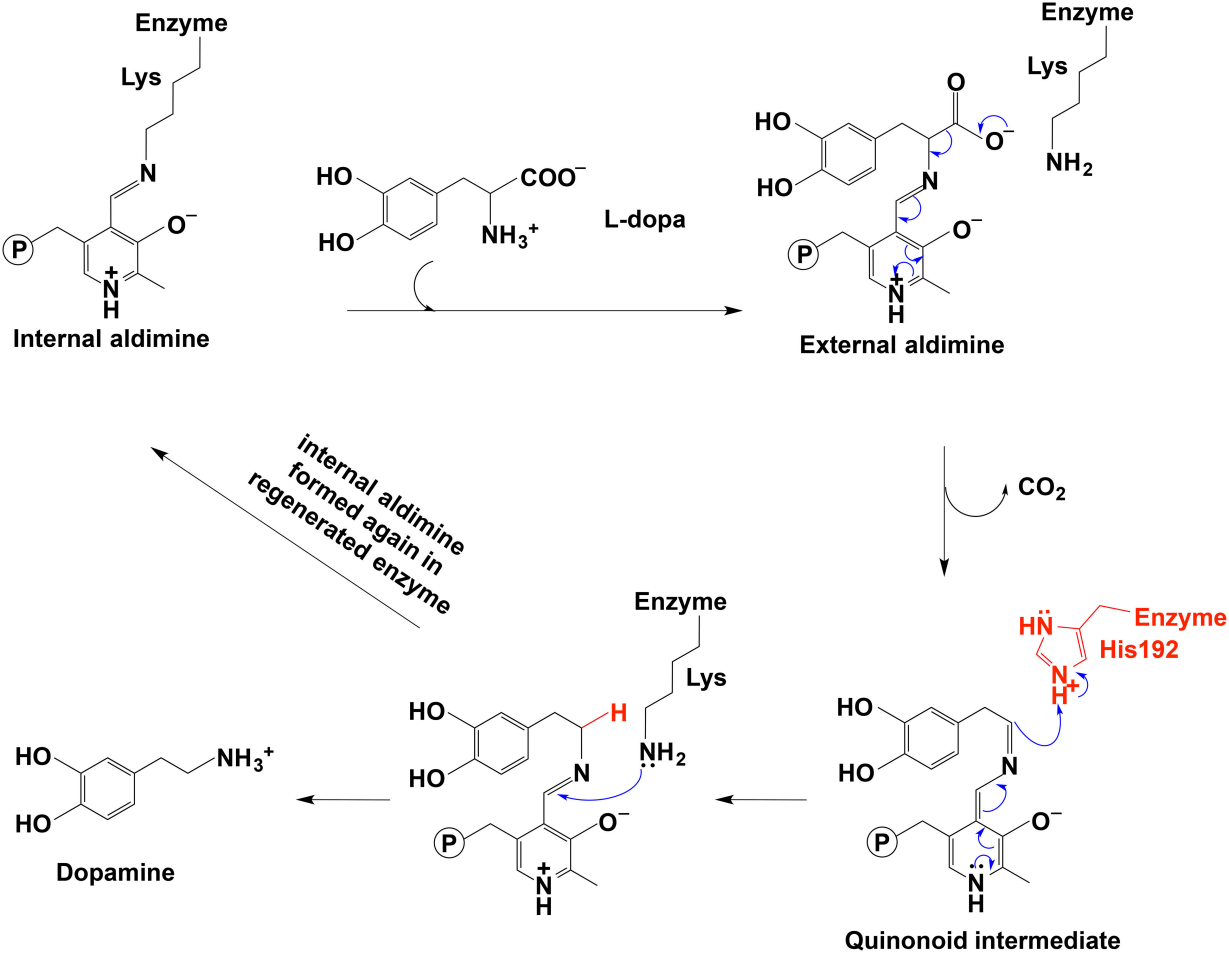
The mechanistic role of His192 residue in typical decarboxylation is shown (Liang et al., 2017).

In the decarboxylation pathway, production of the quinonoid intermediate is reasonable and well accepted. The protonation of quinonoid intermediate at Cα is necessary after decarboxylation and the residues or other factors that promote protonation of Cα have not been determined (at least not clearly specified). Our recent study on 3,4-dihydroxyphenylalanine decarboxylase (DDC) identified that His192 was the residue that protonated quinonoid intermediate after decarboxylation (Liang et al., 2017). This active site histidine residue is stringently conserved in all PLP-dependent aromatic amino acid decarboxylases (Figure 4) and in close proximity with PLP ring and Schiff base structure (Figure 4). After decarboxylation, the carbanion intermediate or quinonoid intermediate (resonance structure of carbanion) is stabilized by the PLP electron sink through electron delocalization. The protonation of Cα by His192 promotes electron shift of the quinonoid intermediate and formation of a new double bond between N atom and C4′. After protonation, the intermediate undergoes transaldimination with the ε-NH_3_ group of active site Lys residue to release dopamine and regenerate PLP-enzyme complex (internal aldimine). The high efficiency of His192 on promoting protonation of Cα is likely the most important step for the intermediates going through the typical DDC-mediated process (Figure 5) (Liang et al., 2017). Mutation of His192 to asparagine resulted in a great decrease in its decarboxylase activity (Liang et al., 2017), which supports its proposed role in decarboxylase reactions. The H192N mutation of DDC decreased V_max_ (285 nmol min^−1^ mg^−1^) of dopamine formation around 11-fold compared with that of the wild-type enzymes (3,357 nmol min^−1^ mg^−1^) (Liang et al., 2017). H192W mutation also lowered the catalytic efficiency of decarboxylation for both 3,4-dihydroxyphenylalanine and 5-hydroxytryptophan (from 128.2 to 21.2 min^−1^mM^−1^ for 3,4-dihydroxyphenylalanine, from 157 to 29.4 min^−1^mM^−1^ for 5-hydroxytryptophan) (Han et al., 2010b).

Other residues involved in substrate binding were also analyzed. In pig kidney DDC complex structure, Thr82 interacts with the 4-catecholhydroxyl group of the carbidopa (a substrate-like inhibitor) (Burkhard et al., 2001). Structural alignment of pig kidney and *Drosophila melanogaster* 3,4-dihydroxyphenylalanine decarboxylases and *Drosophila melanogaster* tyrosine decarboxylase 1 and tyrosine decarboxylase 2 indicated that at the equivalent position with Thr82, Thr was found in pig kidney and *Drosophila melanogaster* 3,4-dihydroxyphenylalanine decarboxylase while *Drosophila melanogaster* tyrosine decarboxylase 1 and tyrosine 2 had a Ser and Ala residue at the equivalent position (Han et al., 2010b). Thr82 appeared to be a potential residue involved in 3,4-dihydroxyphenylalanine binding while Ser or Ala at the equivalent position might be a possible residue for recognizing tyrosine binding. Site-directed mutation of Thr82 to Ser or Ala increased the K_m_ (K_m_ of T82A = 5.6 mM, K_m_ of T82S = 4.5 mM) toward 3,4-dihydroxyphenylalanine by more than 2-folds compared with that of the wild-type enzyme (K_m_ = 2.2 mM). The T82A variant also increased K_m_ for 5-hydroxytryptophan from 0.4 to 3.5 mM. The increased K_m_ values suggested that the binding affinity was lowered by mutation and Thr82 was involved in substrate binding. However, T82S or T82A mutation did not allow the enzyme to be active on tyrosine as speculated from structural alignment result and Thr82 is not the residue to determine the substrate selectivity toward 3,4-dihydroxyphenylalanine rather than tyrosine (Han et al., 2010b).

A bacteria aromatic amino acid decarboxylase (Gene locus tag: PP_2552), which shows high specificity for 3,4-dihydroxyphenylalanine, has been identified from *Pseudomonas putida* KT2440 (Koyanagi et al., 2012). This aromatic amino acid decarboxylase has a higher identity with eukaryotic aromatic amino acid decarboxylases than that with bacteria enzymes. The enzyme catalyzes decarboxylation of 3,4-dihydroxyphenylalanine and 5-hydroxytryptophan with around 200-fold higher catalytic efficiency for 3,4-dihydroxyphenylalanine (k_cat_/K_m_ values for 3,4-dihydroxyphenylalanine and 5-hydroxyphenylalanine are 21 and 0.1 mM^−1^s^−1^ respectively). The decarboxylation activity using phenylalanine, tyrosine, tryptophan, and 3-methoxy-tyrosine as substrates is negligible (Koyanagi et al., 2012). Due to its highest activity on 3,4-dihydroxyphenylalanine, this *Pseudomonas putida* enzyme was proposed as 3,4-dihydroxyphenylalanine decarboxylase (DDC). The k_cat_ value (1.8 s^−1^) on 3,4-dihydroxyphenylalanine is similar to those of mammalian aromatic amino acid decarboxylase (k_cat_ values of hog kidney and rat aromatic amino acid decarboxylase are 8 and 5 s^−1^, respectively) and *Drosophila melanogaster* 3,4-dihydroxyphenylalanine decarboxylase (k_cat_ is 4.7 s^−1^) (Christenson et al., 1970; Hayashi et al., 1993; Han et al., 2010b; Koyanagi et al., 2012). The K_m_ values of the *Pseudomonas putida* (K_m_ = 0.092 mM) and mammalian aromatic amino acid decarboxylases (K_m_ = 0.19 mM for hog kidney enzyme, K_m_ = 0.086 mM for rat enzyme) are in the similar range, but the insect 3,4-dihydroxyphenylalanine decarboxylase K_m_ value is bigger (Christenson et al., 1970; Hayashi et al., 1993; Han et al., 2010b; Koyanagi et al., 2012) (Table 1). Different from mammalian and insect enzymes, which catalyze decarboxylation of both 5-hydroxytryptophan and 3,4-dihydroxyphenylalanine at similar catalytic efficiency level, this *Pseudomonas putida* DDC has high catalytic efficiency (k_cat_/K_m_ = 21 mM^−1^s^−1^) on 3,4-dihydroxyphenylalanine and the catalytic efficiency on 5-hydroxytryptophan is quite low (k_cat_/K_m_ = 0.1 mM^−1^s^−1^) (Gene locus tag: PP_2552) (Christenson et al., 1970; Hayashi et al., 1993; Jebai et al., 1997; Han et al., 2010b; Koyanagi et al., 2012). The activity on tyrosine is also negligible (Koyanagi et al., 2012), which is also in contrast to the similar activity level toward both tyrosine and 3,4-dihydroxyphenylalanine in plant 3,4-dihydroxyphenylalanine/tyrosine decarboxylases (Facchini and De Luca, 1995; Facchini et al., 2000). The substrate specificity is quite distinct from other bacteria aromatic amino acid decarboxylases characterized to date, which are specific for tyrosine (Moreno-Arribas and Lonvaud-Funel, 2001; Connil et al., 2002). Koyanagi et al. compared the mammalian, plant, insect and bacteria aromatic amino acid decarboxylase sequences and found that most of the residues involved in substrate binding in hog kidney aromatic amino acid decarboxylase were identical or biochemically similar in all aligned sequences (Burkhard et al., 2001; Koyanagi et al., 2012). Phe103 is conserved in mammalian, insect and plant aromatic amino acid decarboxylases, which are active on 3,4-dihydroxyphenylalanine/5-hydroxytryptophan (mammalian and insect enzymes), 3,4-dihydroxyphenylalanine/tyrosine (plant enzymes), or phenylalanine/tryptophan (plant enzymes). Instead of a Phe residue, Leu is at the equivalent position in two bacteria decarboxylases, both of which are mainly active on 3,4-dihydroxyphenylalanine (Koyanagi et al., 2012). Leu103 was mutated to Phe in *Pseudomonas putida* 3,4-dihydroxyphenylalanine decarboxylase and the specific activity for both 5-hydroxytryptophan and 3,4-dihydroxyphenylalanine was reduced by 16–17-folds. Based on the results, it is hard to conclude the critical residues involved in substrate preference for 3,4-dihydroxyphenylalanine (Koyanagi et al., 2012).

**Table 1:** Kinetic parameters of *Pseudomonas putida* and mammalian aromatic amino acid decarboxylases (AAAD) and insect 3,4-dihydroxyphenylalanine decarboxylase (DDC) for the two natural substrates L-3,4-dihydroxyphenylalanine (L-dopa) and 5-hydroxytryptophan (5-HTP).

**AAAD or DDC from different organisms**	**Substrates**	**K_**m**_ (mM)**	**k_**cat**_ (s^**−1**^)**	**k_**cat**_/K_**m**_ (mM^**−1**^s^**−1**^)**	**References**
*Pseudomonas putida*	L-dopa	0.092	1.8	21	Koyanagi et al., 2012
AAAD	5-HTP	0.93	0.095	0.1	
Hog kidney AAAD	L-dopa	0.19	8.0	42	Data from Christenson et al. (1970) and k_cat_ values were calculated by Koyanagi et al. (2012)
	5-HTP	0.1	0.77	7.7	
Rat liver AAAD	L-dopa	0.086	5	58	Hayashi et al., 1993
Rat recombinant AAAD with His-Tag	L-dopa	0.14	7	50	Data from Jebai et al. (1997), k_cat_ values were calculated based on the V_max_ values with the reported 50 kDa molecular mass
	5-HTP	0.066	1.5	23	
*Drosophila melanogaster* DDC	L-dopa	2.2	4.7	2.1	Han et al., 2010b
	5-HTP	0.4	1.0	2.5	

From that sequence alignment result (Koyanagi et al., 2012), we also found the critical residue His192 (residue number is from *Drosophila melanogaster*) (Liang et al., 2017) was conserved at the equivalent position in this bacteria decarboxylase (Koyanagi et al., 2012) and other aromatic amino acid decarboxylases. However, the residues involved in different substrate specificity of aromatic amino acid decarboxylases have not been fully elucidated.

#### Racemase

Racemases catalyze deprotonation of Cα to form a carbanionic intermediate and then reprotonation of Cα occurs on either *re*-face or *si*-face of the intermediate to make the stereochemical inversion of molecules in both directions. Racemases can be divided into two classes, PLP-dependent amino acid racemases and PLP-independent racemases (Conti et al., 2011). PLP-dependent racemases include serine racemase, alanine racemase and aspartate racemase in eukaryotes and alanine racemase, serine racemase and arginine racemase in bacteria.

**Figure 6:**
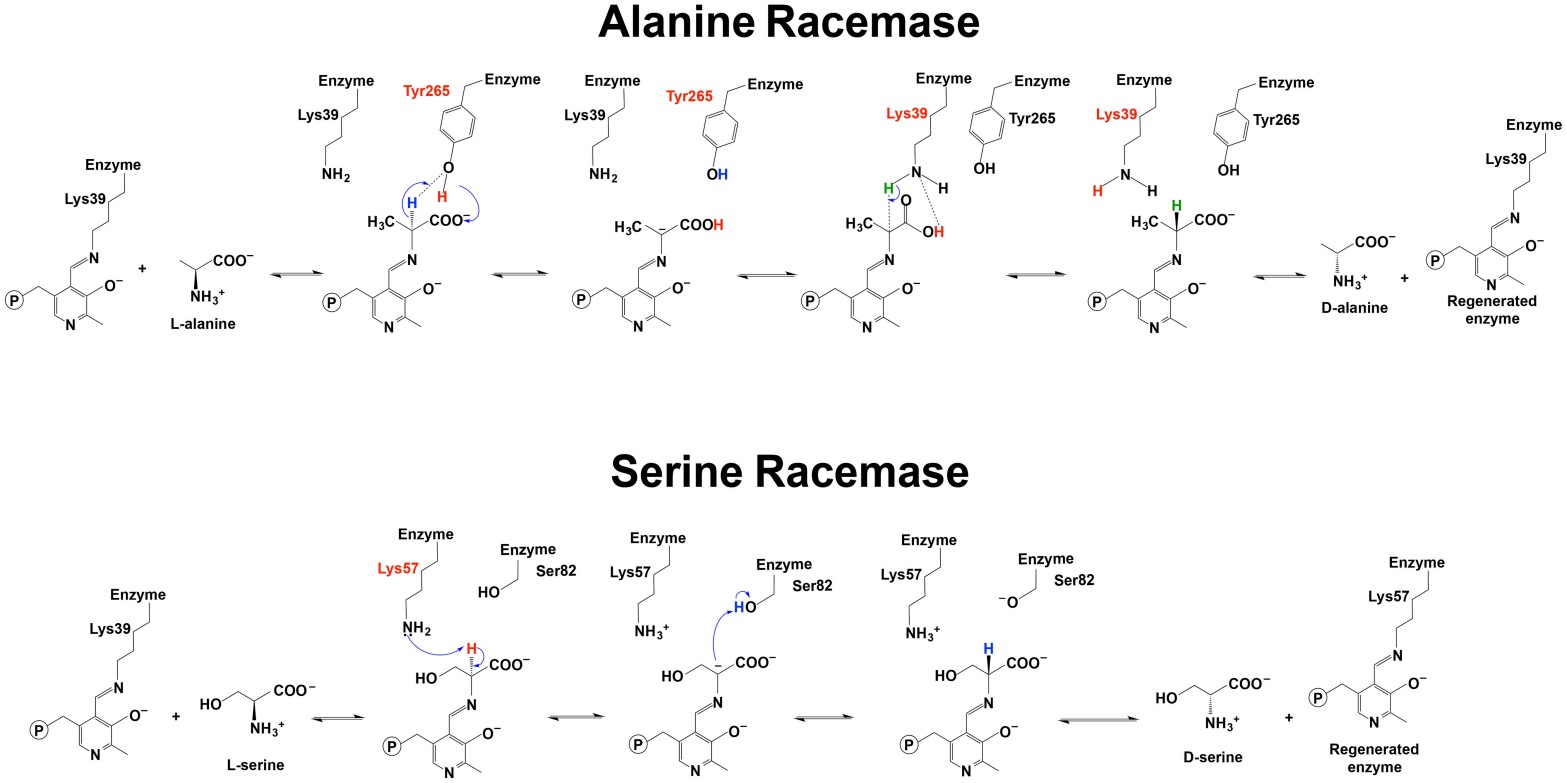
Reaction mechanisms of alanine racemase and serine racemase (Watanabe et al., 1999, 2002; Yoshimura and Goto, 2008; Goto et al., 2009).

For alanine racemase, it has been proposed that Tyr265 was the residue functioning in deprotonation at Cα of L-alanine. The Tyr265 side chain was predicted to be the ionic form when the racemase-catalyzed reaction was under the most active condition (pH 7–9) (Watanabe et al., 1999; Spies and Toney, 2003; Spies et al., 2004; Strych et al., 2007; Yoshimura and Goto, 2008). The theoretical model of Ondrechen et al. also seemed to support the role of Tyr265 in deprotonation (Ondrechen et al., 2001). The Schiff base Lys39 residue functioned to reprotonate the intermediate for the conversion from L-alanine to D-alanine (Figure 6) (Watanabe et al., 1999; Strych et al., 2007; Yoshimura and Goto, 2008). For conversion from D-alanine to L-alanine, Lys39 functioned to deprotonate and Tyr265 reprotonated. Based on these results, the pKa of the external aldimine was estimated to be around 9, which was between pKa values of Lys39 and Tyr265 (Spies and Toney, 2003; Spies et al., 2004).

The role of Tyr265 residue as a general acid-base for L- to D-alanine or D- to L-alanine conversion (Watanabe et al., 1999; Spies and Toney, 2003; Spies et al., 2004; Strych et al., 2007; Yoshimura and Goto, 2008) was reported to need a nearby His166 residue to form hydrogen bond and lower the pKa of Tyr265 side chain (Seebeck and Hilvert, 2003). Seebeck and Hilvert suggested that the Tyr265 to Ala mutation of *Geobacillus stearothermophilus* alanine racemase generated new aldolase activity while the racemization activity was largely reduced. Simultaneously, the substrate selectivity was also changed and the Y265A mutation of alanine racemase allowed the enzyme to be active on D-β-hydroxy-α-amino acid because Tyr to alanine mutation made the active site bigger. The single mutation of the alanine racemase with generated aldolase activity and changed substrate specificity is a good example of how PLP-dependent reaction specificity is achieved through crucial active site residue (Seebeck and Hilvert, 2003).

For serine racemase, Lys57 and Ser82 were proposed to be the residues functioning as a general acid-base in the reversible reaction catalyzed by *Schizosaccharomyces pombe* serine (PDB: 1WTC) (Figure 6) (Goto et al., 2009). Lys56 and Ser84 were reported to be at the equivalent position with yeast Lys57 and Ser82 in human and rat serine racemase (PDB: 3L6B, 3L6R, 3L6C, and 3HMK) (Smith et al., 2010). Compared with the Lys residue, Ser82 needs a pKa low enough to function as a general acid-base. A recent study on human serine racemase suggested that Lys114 and one water molecule might work together to lower pKa of Ser84 through a hydrogen bond network among the Ser84, Lys114, and a water molecule (Nelson et al., 2017).

It was also reported that a conformational change of the enzyme contributed to the proper orientation of the Ser residue to function as a general acid/base. After substrate binding, a conformational change of the small domain to close the active site was observed in *Schizosaccharomyces pombe* serine racemase (Goto et al., 2009). Some structural analyses of mammalian serine racemase identified a small domain movement induced by the substrate or inhibitor binding to close the active site. Intriguingly, the shift of the small domain toward the active site contributed to the specific orientation of Ser84 residue (at the equivalent position with Ser82 in PDB: 1WTC) for the reprotonation step to convert L-serine to D-serine (Smith et al., 2010).

Nitoker and Major performed the multiscale quantum-classical simulations and analyzed the free energy profile of the racemization catalyzed by human and rat serine racemase in both gas phase and aqueous solution. Their results suggested a stepwise reaction mechanism of serine racemase, which was similar to that of alanine racemase. Their analyses indicated that it was the solvation effects and the long-range electrostatic interactions with the active site residues that mainly stabilized the intermediate formed during reaction. This conclusion was evidenced by the free energy profile that the deprotonation was unfavored in a gas phase while the aqueous solution stabilized the intermediate formed after first step deprotonation. The active site residues Asn86, His87, and Ser83 functioned to lower the electrostatic repulsion between the negative charges generated by deprotonation step and the carboxylate group (Nitoker and Major, 2015). The authors identified that the unprotonated pyridine ring N seemed to be the correct form for the racemization reaction catalyzed by serine racemase because protonation of the pyridine ring N might facilitate α,β-elimination reaction of L- or D-serine to produce ammonia and pyruvate, which was another activity of serine racemase (Nitoker and Major, 2015). To keep the pyridine ring N from being protonated, serine racemase (fold type II) has one Ser residue that forms a neutral hydrogen bond with pyridine ring N (Jhee et al., 1998; Goto et al., 2009) while bacteria alanine racemase (fold type III) has one Arg residue at the equivalent position (Canu et al., 2014). The study on R219E variant of alanine racemase also identified the possibility of side reactions when the pyridine ring N was protonated in the mutated enzyme (Rubinstein and Major, 2010).

#### PLP-Dependent Aldolase

Aldolase catalyzes the aldol condensation, leading to C-C bond formation in a stereoselective way. Based on the mechanism and substrate specificity, the aldolases include lysine-dependent, metal-dependent, and PLP-dependent aldolases (Contestabile et al., 2001; Dean et al., 2007; Falcicchio et al., 2014). PLP-dependent aldolases have been classified into serine hydroxymethyltransferase (catalyzing glycine formation from L-serine, and 5,10-methylene-tetrahydrofolate formation from tetrahydrofolate) (Fujioka, 1969) and threonine aldolases (mediating the reversible reaction for condensation of small amino acids and aldehyde or degradation of threonine, e.g., condensation of glycine and acetaldehyde to threonine or threonine degradation to acetaldehyde and glycine) (Bell and Turner, 1977; Fesko, 2016).

Compared with D-threonine aldolases, there are more L-threonine aldolases being identified. Co-crystallization (PDB: 1LW4) of *Thermotoga maritime* L-threonine aldolase with L-*allo*-Thr and models built with L-Thr suggested that for L-*allo*-Thr substrate, His83 was the prospective residue serving as a general base in catalysis to accept one proton from the substrate, but for L-Thr cleavage, it was predicted that, instead of His83, the general base was His125 (from another subunit) or a water molecule (Kielkopf and Burley, 2002). The existence of His83 and His125 might allow flexibility using both L-*allo*-Thr and L-Thr as substrates. Tyr87 was the residue leading to the substrate preference for L-*allo*-Thr over L-Thr and the residue at position 87 or equivalent was the only residue varied in all threonine aldolases (Tyr or Phe in enzymes with substrate preference for L-allo-Thr) involved in stereospecificity. It was proposed that the larger side chain of residue at position 87 corresponds to a higher preference for the *allo* isomer (Kielkopf and Burley, 2002). The native and complex structures (PDB: 4LNJ, 4LNM, 4LNL) of *Escherichia coli* L-threonine aldolase revealed that the substrate binding had little effect on the structure, and His83 and His126 (at equivalent positions with His83 and His125 in *Thermotoga maritime* L-threonine aldolase) interacted with a water molecule and the substrate hydroxyl group through hydrogen bonds. A single mutation of either residue and double mutation of both greatly reduced the k_cat_ of the enzyme, but none of these mutations could remove all the activity. His83 to Asn or Phe mutation showed no activity unless a high concentration of PLP was added because His83 was involved in PLP stacking. His126 to Asn or Phe mutation actually doubled the k_cat_ with *erythro* and *threo* isomers of threonine as substrates. These results suggested that the two histidine residues might not be directly finctioning as a general acid/base. Due to no other active site residues near substrate hydroxyl group to abstract a proton, these authors hypothesized that His83 and His126 might play a role to help with the coordination of one water molecule, and this water molecule was the one functioning as a general base by transferring a proton to the phosphate group through hydrogen bond (Di Salvo et al., 2014). The mutational study of residue at position 87 was also conducted in *Escherichia coli* L-threonine aldolase (Di Salvo et al., 2014). Different from the proposed role of residue 87 in *Thermotoga maritime* L-threonine aldolase (Kielkopf and Burley, 2002), *Escherichia coli* L-threonine aldolase F87A mutation, with a reduced side chain size at position 87, did not change substrate preference. Another threonine aldolase from *Pseudomonas aeruginosa*, which does not have any substrate preference, has one Asp residue at position 87. However, when *Escherichia coli* L-threonine Phe87 was mutated to Asp, with reduced side chain bulk, the substrate preference for L-*allo*-threonine was even increased. Based on these mutations at position 87, Di Salvo et al. believed that it was the overall active site microenvironment, instead of specific residues that affected the substrate preference (Di Salvo et al., 2014). The role of water molecule as a weak base was also hypothesized in the reaction mechanism of D-threonine aldolase. A crystal structure of a D-threonine aldolase from *Alcaligenes xylosoxidans* showed that this PLP-dependent enzyme had one metal binding in the active site. The authors proposed that this D-threonine aldolase was similar to L-threonine aldolase in having a water molecule to mediate deprotonation. The metal ion bound the substrate β-hydroxy group and might activate and coordinate the group for deprotonation by another conserved residue His193 (Uhl et al., 2015).

In terms of the donor selectivity and substrate binding, although both His83 and His126 residues might not function as general acid/base directly, they were involved in substrate binding and stereospecificity in *Escherichia coli* L-threonine aldolase. Both H83F and H126F variants increased the stereospecificity toward L-threonine (Di Salvo et al., 2014). In *Aeromonas jandaei* L-threonine aldolase, the substrate specificity was affected by His85 (at the equivalent position as His83 in *Escherichia.coli* L-threonine aldolase), Tyr89, His128 (at the equivalent position as His126 in *Escherichia coli* L-threonine aldolase), Glu90 and Asp126 (Qin et al., 2014).

Threonine aldolases have been known previously accepting glycine as a donor, but analyses of the expressed L-threonine aldolases and D-threonine aldolases identified some threonine aldolases with novel and broader donor specificities. For example, L-threonine aldolases and D-threonine aldolases from *Aeromonas jandaei* and *Pseudomonas sp*. respectively, could accept D-Ser, D-Ala, and D-Cys as donors (Fesko et al., 2009). Fesko et al. attempted to analyze residues important for donor specificity of *Aeromonas jandaei* threonine aldolases. The region Met281 to Arg313 seemed undergoing some conformational changes and was suggested to be involved in substrate specificity and active site flexibility. Arg313 was a residue conserved in all L-threonine aldolases to contribute to the donor carboxylate group stabilization. Intriguingly, Arg313 showed slight differences of the spatial orientation in various threonine aldolases, suggesting a potential role in affecting the hydrogen bond network between the active site environment and the donor substrate. These interactions were suggested as being critical for the donor substrate specificity through stabilizing the donor in an active orientation for catalysis (Fesko et al., 2015).

For the donor specificity of another group of PLP-dependent aldolase, sequence alignment of serine hydroxymethyltransferase and its analog, α-methylserine hydroxymethyltransferases, indicated that Tyr55 and Tyr65 in serine hydroxymethyltransferases, and Thr60 and His70 in α-methylserine hydroxymethyltransferases were residues involved in donor specificity. Mutation of Tyr55 to Thr in serine hydroxymethyltransferase enabled the enzyme to use both D-Ala and D-Ser while the wild-type enzyme accepted glycine and D-Ala, although the activity of wild-type enzyme using D-Ala was hard to be detected due to the side reaction (Shostak and Schirch, 1988; Hernandez et al., 2015).

#### Cystathionine β-Synthase

Cystathionine β- and γ-synthase are PLP-dependent enzymes involved in H_2_S formation and also two critical enzymes in the trans-sulfuration pathway (Singh et al., 2009). Cystathionine β-synthase catalyzes β-replacement reactions using cysteine or serine and homocysteine as substrates to produce cystathionine and H_2_S or water, respectively (Koutmos et al., 2010).

Characterization of *Lactobacillus plantarum* cystathionine β-synthase identified that this wild-type enzyme showed both *O*-acetyl-L-serine-dependent cystathionine β-synthase activity and *O*-acetyl-L-serine sulfhydrylase activity (formation of L-cysteine from *O*-acetyl-L-serine and H_2_S) (Matoba et al., 2017). The existence of both activities is probably due to higher similarity in primary sequence and active site conformation with bacteria *O*-acetyl-L-serine sulfhydrylase than that with cystathionine β-synthase (Hullo et al., 2007). For substrate preference, H_2_S formation catalyzed by the *Lactobacillus plantarum* cystathionine β-synthase was greatly enhanced by using both L-cysteine and L-homocysteine as substrates as compared with the H_2_S produced in the absence of L-homocysteine. With the presence of L-homocysteine, the k_cat_/K_m_ for H_2_S synthesis was one magnitude higher than that without L-homocysteine. L-cystathionine and L-lanthionine were major and minor byproduct, respectively, when H_2_S was formed (Matoba et al., 2017).

Structural analyses and mutation study suggested that conserved Ala70 and Glu223 in the active site were residues important for H_2_S formation. Glu233 has a restricted conformation and regulates the substrate-binding site size by interacting with residues in another domain. Ala70 and Glu223 are conserved in cystathionine β-synthase while Ser and Gly are conserved at the equivalent positions in bacteria type A *O*-acetyl-L-serine sulfhydrylase. A70S variant seemed to improve the lifetime of aminoacrylate intermediate and inhibited the localization of the nucleophilic atom to the catalytically active position. E223G variant showed a decreased k_cat_ in β-replacement reaction, which might be due to a narrower substrate-binding site inhibiting product releasing in the E233G variant (Matoba et al., 2017). *Bacillus anthracis* cystathionine β-synthase is active to *o*-acetylserine, cysteine and homocysteine, but not active to serine. A mutational study of *Bacillus anthracis* cystathionine β-synthase indicated the importance of conserved Glu220 for enzyme activity to produce H_2_S (Devi et al., 2017). Other key residues were proposed by the analyses of human cystathionine β-synthase. PLP is anchored in the active site through residue Lys88. The structure of cystathionine β-synthase showed that the Lys88 residue was close to substrate Cα, Schiff base imino nitrogen and C4A atom. The structure of intermediates indicated that Lys88 might have a role in deprotonation of Cα as a general base to activate its substrate. It was postulated that Lys88 contributed to the stabilization of carbanionic intermediate through electrostatic interactions between the ε-${\text{NH}}_{3}^{+}$ of Lys88 and Cα (Cα had some negative charge characteristics in the intermediate) (Koutmos et al., 2010). A Gly307 residue in human cystathionine β-synthase was predicted to be important for forming the catalytically active conformation state(s) of Tyr308 in the intermediate generation. G307S variant showed no detectable activity (Gupta et al., 2018).

It was also reported that cystathionine β-synthase activity might be regulated through different mechanisms. Koutmos et al. analyzed the structure, especially the regulatory and catalysis modules, of *Drosophila melanogaster* cystathionine β-synthase with two intermediates captured and suggested one allosteric activation mechanism for the enzyme (Koutmos et al., 2010). Based on the structure of the activated enzyme, the authors proposed that *S*-adenosylmethionine binding activated the enzyme by restricting the conformational flexibility of the regulatory domain to enhance the active site access and stabilize the PLP binding site by introducing changes into a linker region involved in PLP binding (Koutmos et al., 2010). In addition to the allosteric mechanism, a study on human cystathionine β-synthase indicated another activity regulation mechanism, which was through the reducing status of the disulfide bond in the ^272^CXXC^275^ motif. This motif was in the central domain of human cystathionine β-synthase with either oxidized or reduced states, and the activity of reduced state was higher (2–3-fold) (Niu et al., 2018).

#### O_2_-Using PLP-Dependent Enzymes

PLP-dependent oxidative reactions are not common, although some PLP-dependent enzymes have been reported to catalyze O_2_-involved oxidation side reactions, e.g., dopa decarboxylase. Our recent study indicated that a number of insect PLP-containing proteins, listed as aromatic amino acid decarboxylases or aromatic amino acid decarboxylase-like enzymes in databases, actually catalyzed the decarboxylation-oxidative deamination reaction of aromatic amino acids to their aromatic acetaldehydes (Vavricka et al., 2011; Liang et al., 2017). Similar proteins were also detected in plants (Kaminaga et al., 2006; Torrens-Spence et al., 2012, 2013, 2014). These proteins share high sequence similarity or identity (~50%) to their respective insect and plant aromatic amino acid decarboxylases, which explains why these proteins have been classified as aromatic amino acid decarboxylases. Our previous studies indicated that these enzymes catalyzed decarboxylation-oxidative deamination to produce the corresponding aromatic acetaldehyde, CO_2_, H_2_O_2_, and NH_3_ (Vavricka et al., 2011; Torrens-Spence et al., 2013, 2014; Liang et al., 2017). Based on a key role of the aromatic acetaldehyde in insect cuticle formation, the enzyme was arbitrarily named aromatic acetaldehyde synthase, but the mechanism or pathway from aromatic amino acids to their corresponding aromatic acetaldehyde derivatives is unclear (Vavricka et al., 2011; Liang et al., 2017).

**Figure 7:**
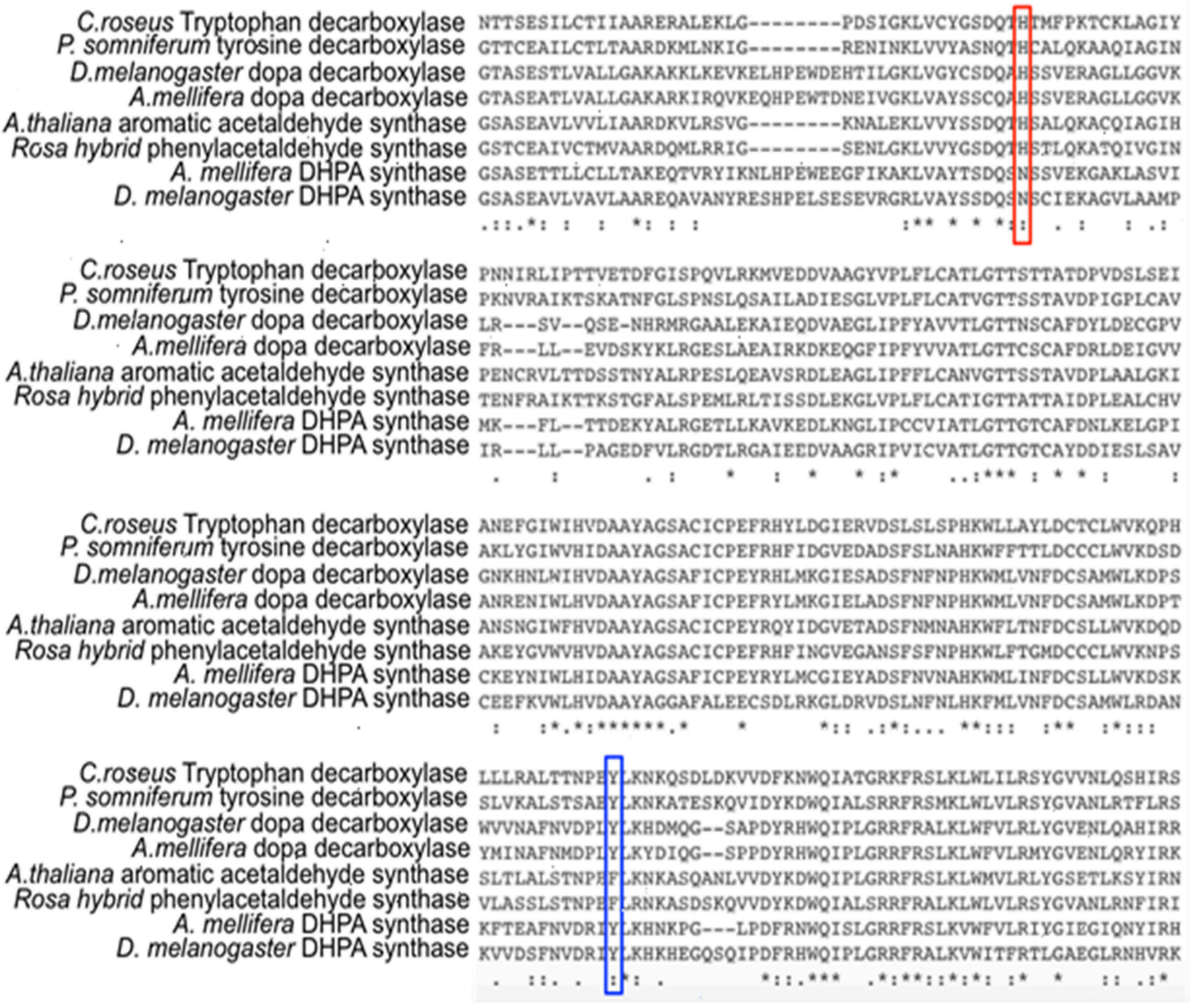
Partial protein sequence alignment of plant and insect decarboxylases and aromatic acetaldehyde synthases. *Catharanthus roseus* tryptophan decarboxylase (P17770), *Papaver somniferum* tyrosine decarboxylase (AAC61842), *Drosophila melanogaster* dopa decarboxylase (NP_724164.1), *Apis mellifera* dopa decarboxylase (XP_394115.2), *Arabidopsis thaliana* aromatic acetaldehyde synthase (NP 849999), *Rosa hybrid cultivar* phenylacetaldehyde synthase (ABB04522.1), *Apis mellifera* 3,4-dihydroxyphenylacetaldehyde (DHPA) synthase, *Drosophila melanogaster* DHPA synthase (NP_724162.1) protein sequences are compared. The conservations of His vs. Asn residues at the equivalent position with His192 of *Drosophila melanogaster* dopa decarboxylase (NP_724164.1) are highlighted by red box. The conservations of flexible loop Tyr vs. Phe at the equivalent position with Tyr348 of *Catharanthus roseus* tryptophan decarboxylase (P17770) are highlighted by blue box.

**Figure 8:**
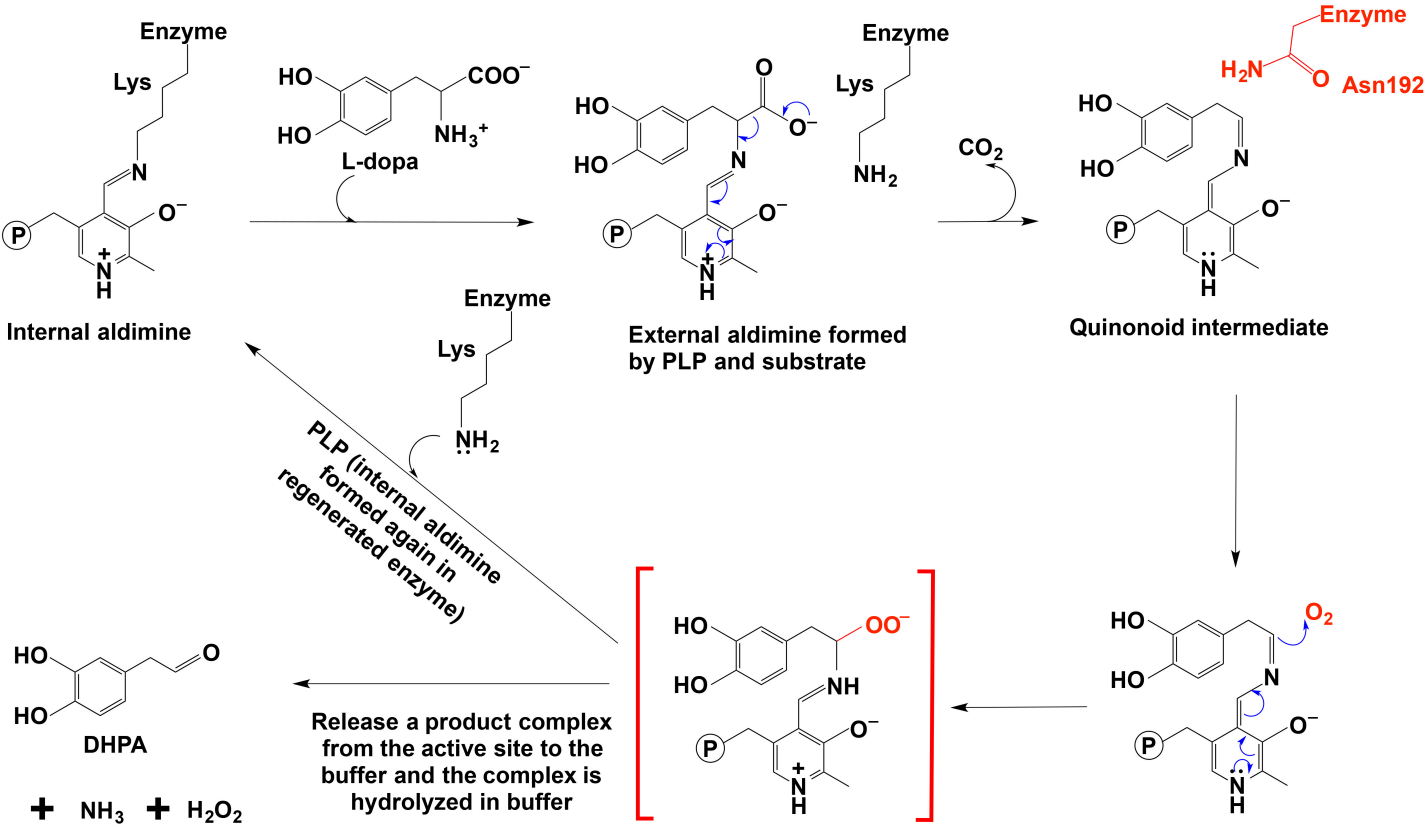
The mechanistic role of Asn192 residue in *Drosophila melanogaster* DHPA synthase (Liang et al., 2017).

Comparative studies on primary structures between identified insect aromatic amino acid decarboxylases and aromatic acetaldehyde synthases showed no apparent clues to tell them apart, but a comparison of 3-dimensional structures or model structures, particularly their active site residues, identified some differences in residues between two enzymes (Liang et al., 2017). In aromatic amino acid decarboxylases, a histidine residue was determined to play an essential role in protonation of carbanionic intermediate, which was considered as a necessary step toward final product formation (Liang et al., 2017). In insect aromatic acetaldehyde synthases, an asparagine residue occupies the similar position (Figure 7). Although aromatic acetaldehyde synthase is also able to catalyze decarboxylation, the rapid protonation of the intermediate becomes problematic. The quinonoid intermediate, once formed, is likely quite reactive, but the absence of the histidine residue prevents its rapid protonation at Cα, which leaves opportunity for oxygen attack (Figure 8) (Liang et al., 2017). This likely increase the life time of the intermediate and gives opportunity for oxygen attack. Instead of protonation and rapid hydrolysis to form dopamine, the rate of the overall reaction of aromatic acetaldehyde synthase slows down greatly with a V_max_ of 270 nmol min^−1^ mg^−1^, which is in contrast with the aromatic decarboxylase with a V_max_ of 3,357 nmol min^−1^ mg^−1^ and with the similar level of K_m_ values on the same substrate (3,4-dihydroxyphenylalanine) (Liang et al., 2017). After oxygen addition, the substrate complex likely is released from the enzyme active site and disintegrates rapidly in a buffer environment (Figure 8).

The proposed mechanism was supported by site-directed mutagenesis, in which a mutation of N192 to H192 in a *Drosophila melanogaster* 3,4-dihydroxyphenylacetaldehyde (DHPA) synthase led to the mutated enzyme with considerable decarboxylase activity and a mutation of H192 to N192 in *Drosophila melanogaster* 3,4-dihydroxyphenylalanine decarboxylase (DDC) made the mutated DDC have a DHPA synthase activity comparable to the DHPA synthase wild type in magnitude (Liang et al., 2017). Both N192H DHPA synthase variant and H192N DDC variant have not greatly affected substrate affinity as indicated by K_m_ values. K_m_ of DHPA synthase N192H variant increased from 0.88 to 1.23 mM, and K_m_ of DDC H192N variant decreased from 1.26 to 0.82 mM. The N192H DHPA synthase variant decreased the DHPA synthase activity (from 270 to 163 nmol min^−1^ mg^−1^), but increased the dopamine formation activity (decarboxylase activity) from a negligible level to one of the two major activities (V_max_ of decarboxylase activity was 78 nmol min^−1^ mg^−1^). DDC H192N variant decreased decarboxylase activity (V_max_ from 3357 to 285 nmol min^−1^ mg^−1^) and increased the DHPA synthase activity from barely detectable (no more than 1% of the decarboxylase activity) to comparable to that of wild-type DHPA synthase on magnitude (V_max_ is 163 vs. 270 nmol min^−1^ mg^−1^ of wild-type DHPA synthase) (Liang et al., 2017).

In DDC-catalyzed reactions, formation of a small amount of DHPA (~0.3%) has been reported (Liang et al., 2017), suggesting that the proposed quinonoid intermediate is intrinsically reactive and presence of His residue, capable of rapidly protonating the intermediate, is unable to have all intermediate molecules undergoing protonation and subsequent hydrolysis to amine.

Different from insect aromatic acetaldehyde synthases, plant enzymes have His residue conserved in both decarboxylase and aromatic acetaldehyde synthase, but the plant enzymes have a conserved tyrosine or phenylalanine in a flexible loop region functioning to discriminate typical decarboxylation and phenylacetaldehyde synthesis reaction, respectively. Although tyrosine residue in plant decarboxylases may not be able to directly protonate the intermediate, it can stabilize the proton transfer process from histidine residue to Cα after decarboxylation. In contrast, phenylalanine has no stabilizing effect for proton transfer, which leaves the intermediate being attacked by oxygen (Torrens-Spence et al., 2013). The proposed role of the Tyr/Phe residue was supported by site-directed mutagenesis study. Both *Arabidopsis thaliana* aromatic acetaldehyde synthase F338Y variant (K_m_ = 4.6 mM) and *Catharanthus roseus* tryptophan decarboxylase Y348F (K_m_ = 0.095 mM) had little effect on the K_m_ values compared with those of their wild-type enzymes (K_m_ values were 5.1 and 0.12 mM for *A. thaliana* aromatic acetaldehyde synthase (accession: NP_849999) and *C. roseus* tryptophan decarboxylase (accession: P17770), respectively), indicating that both mutations did not apparently change the binding affinity to phenylalanine and tryptophan, respectively (Torrens-Spence et al., 2013). The F338Y variant of *A. thaliana* aromatic acetaldehyde synthase showed decarboxylation activity, which was around 3-fold higher (V_max_ = 341 nmol min^−1^ mg^−1^) than the original acetaldehyde synthase activity (V_max_ = 112 nmol min^−1^ mg^−1^) of its wild-type enzyme (Torrens-Spence et al., 2013). By mutating Tyr348 to Phe in *C. roseus* tryptophan decarboxylase, the decarboxylase enzyme was converted to an aromatic acetaldehyde synthase. The generated acetaldehyde synthesis activity (V_max_ = 160 nmol min^−1^ mg^−1^) on the same substrate was around 17-fold lower than the original decarboxylation activity (V_max_ = 2,710 nmol min^−1^ mg^−1^) of the wild-type enzyme (Torrens-Spence et al., 2013).

Although the presence of phenylacetaldehyde synthases has been clearly established (Kaminaga et al., 2006; Torrens-Spence et al., 2012, 2013; Liang et al., 2017), a number of critical questions remain to be answered. We discussed the involvement of oxygen in somewhat non-enzymatic manner. Oxygen needs to be activated prior to oxidation-reduction processes. However, PLP does not seem to be able on oxygen activation. We propose that the product complex is released first from the active site and then disintegrates to aromatic acetaldehyde, NH_3_, and H_2_O_2_. The disintegration of the complex likely needs acid-base mediated reactions. We proposed the mechanism of this insect phenylacetaldehyde synthase, but the detailed mechanism, including the product complex disintegration, needs to be elucidated. Compared with the enzyme active site environment, acid-base mediated reactions for product complex disintegration may occur easily in a buffer, but the true mechanism may be different from the proposed mechanism. The research on a phenylacetaldehyde synthase from *Petunia hybrida* cv. Mitchell plant suggests a radical mechanism. The K_m_ of *Petunia hybrida* cv. Mitchell phenylacetaldehyde synthase is 1.18 mM (Kaminaga et al., 2006), which is similar to the K_m_ of the aromatic acetaldehyde synthase identified from insect (Liang et al., 2017). The k_cat_ is 0.8 s^−1^ per homotetramer (Kaminaga et al., 2006). The reaction is O_2_-dependent as confirmed by O_2_ consumption. It was suggested that the *Petunia hybrida* cv. Mitchell phenylacetaldehyde synthase catalyzed oxidative decarboxylation through a radical mechanism (Kaminaga et al., 2006), which was in contrast with the usually proposed non-radical mechanism with a non-radical carbanion or quinonoid intermediate. The authors indicated that the isotope labeling results (about half of the deuterium was found in the phenylacetaldehyde product) better supported the radical mechanism than a non-radical mechanism. These authors argued that if the reaction was through a quinonoid intermediate, all the deuterium should be in the phenylacetaldehyde (Kaminaga et al., 2006). The study on *Petunia hybrida* cv. Mitchell phenylacetaldehyde synthase provided some clues for a radical mechanism (Kaminaga et al., 2006) and more detailed studies are needed for further clarifications about aromatic acetaldehyde synthase-mediated reactions.

Other oxidative reactions catalyzed by PLP-dependent enzymes were also reported. Han et al. (2015) reported one PLP-dependent L-arginine α-deaminase, γ-hydroxylase, MppP enzyme (accession number: KDR62041, from *Streptomyces wadayamensis*), could utilize O_2_ as a reagent, and the products formed were 2-oxo-5-guanidinovaleric acid and 2-oxo-4-hydroxy-5-guanidinovaleric acid. MppP enzyme is the first reported PLP-dependent hydroxylase and quite similar to PLP-dependent aromatic amino acid decarboxylases in the function of stabilizing a quinonoid intermediate for reacting with O_2_. There were two quinonoid intermediates formed in the reaction as supported by the absorbance λ_max_ at 510 nm and 560 nm. The first quinonoid intermediate showed a high stability in the reaction condition without O_2._ The K_m_ of MppP enzyme using L-Arg as the substrate was 50.2 μM and k_cat_ was 0.22 s^−1^ (Han et al., 2015). It was hypothesized that the modest activity level might be a reflection that the full activity of MppP might need other enzyme members in MPP biosynthetic cluster, or that the activity of MppP should be similar to that of nonribosomal peptide synthase (with a slow catalysis) associated with MppP (Sun et al., 2014; Han et al., 2015). Their structural analyses and comparison with PLP-dependent aromatic amino acid decarboxylase suggested that the active site Asp218 residue might contribute to phosphate group rotation. Prior to substrate binding, Asp218 was suggested to interact with PLP phosphate group indirectly. After substrate binding, catalytic Lys211 of the enzyme interacted with Asp218 through a hydrogen bond and Asp218 appeared to make the phosphate group rotate out of the PLP ring plane. Another residue His29 might be the key residue for PLP-dependent hydroxylase (Han et al., 2015). Compared with MppP enzyme, decarboxylases have Asn300 (residue number is from pig kidney decarboxylase, PDB: 1JS3; Burkhard et al., 2001) at the equivalent position as Asp218 in MppP and decarboxylases do not have counterpart residue at the equivalent position with His29. There is one Thr82 residue at the approximate position (Burkhard et al., 2001; Giardina et al., 2011; Han et al., 2015). The authors suggested that analyses of the role of Asp218 and/or His29 were being conducted. His29 was unique to MppP and might be the signature residue of PLP-dependent hydroxylases (Han et al., 2015).

Ringel et al. reported one PLP-dependent periplasmic enzyme PvdN that catalyzed the oxidative decarboxylation using O_2_ and glutamic acid as substrates to produce succinamide for the pyoverdine modification (Ringel et al., 2016). This is the first PLP-dependent enzyme that catalyzes the reaction of amide formation after amino acid decarboxylation, O_2_ attack and deprotonation. The hypothesized mechanism of PvdN was that a peroxo intermediate formed at Cα after decarboxylation. This step was followed by proton abstraction at Cα, electron shuffling and product complex disintegration to form corresponding amide and H_2_O. The PvdN crystal structure showed a big channel for the pyoverdine side chain to enter from surface to the active site, and the other small channel for O_2_ and CO_2_ (Ringel et al., 2016). The proposed mechanism of PvdN shares some steps similar to the aromatic acetaldehyde synthase, i.e., decarboxylation and peroxo intermediate formation at Cα, but the key residues that are involved in the amide formation are unknown and need further analyses.

Cap15, a PLP-dependent monooxygenase-decarboxylase, uses O_2_ and (5′S, 6′R)-glycyluridine [(5′S, 6′R)-GlyU] as substrates to produce CO_2_ and uridine-5′-carboxamide (CarU) (Huang et al., 2018). Unlike aromatic acetaldehyde synthase to form H_2_O_2_ as one product, Cap15 catalyzes the incorporation of one O atom into carboxamide containing CarU. Analyses on Cap15 indicated that PLP was the only cofactor for Cap15 to activate O_2_ before decarboxylation. They proposed that the carbanionic intermediate, which was the resonance form of quinonoid intermediate, had a role comparable to that of the reduced FAD for monooxygenase. Due to no detectable CO_2_ formation when O_2_ was excluded from the reaction, it was predicted that O_2_ reduction occured before decarboxylation. After deprotonation, the carbanionic intermediate or quinonoid intermediate was formed, and O_2_ got one electron from the carbanionic intermediate to form a GlyU-PLP radical and superoxide. The superoxide was protonated and rebound with the radical to form a hydroperoxide species. Then decarboxylation occurred, and one O atom from O_2_ was incorporated into the product complex and the other O atom from O_2_ was eliminated into H_2_O. The disintegration of product complex released carboxamide-containing CarU (Huang et al., 2018). The reaction catalyzed by Cap15 is PLP-, O_2_, and phosphate-dependent, but the role of phosphate for the reaction catalyzed by Cap15 is unknown. K_m_ of Cap15 is 560 μM and k_cat_ is 0.93 min^−1^. Protein sequence alignment indicated that Lys230 was a possible residue that formed an internal aldimine with PLP. Lys262, Lys265, and Lys303 were also predicted as potential residues that were important for Cap15 enzyme. The site-directed mutagenesis result showed that K230A and K303A mutations retained the activity, but K262A and K265A showed no detectable activity (Huang et al., 2018). It was indicated that some kinetic analyses of these variants were ongoing and more structural information would provide insights into the role of these residues and other residues critical for catalysis (Huang et al., 2018). Their study provided one possibility for O_2_ activation and should stimulate further research for exploring and verifying the proposed mechanism.

O_2_-using PLP-dependent enzymes have become one emerging field for PLP-dependent enzyme study and many unknown aspects need to be explored. The detailed mechanism for different O_2_-using PLP-dependent enzymes, especially O_2_ activation, requires further elucidation. In addition, the structure-function relationship, e.g., residues involved in substrate specificity and reaction specificity, has not been clearly established and awaits further study after characterization of more reactions catalyzed by O_2_-using PLP-dependent enzymes.

## Summary

PLP-containing enzymes are diverse and catalyze numerous reactions. Toward this end, scientists have learned sufficiently the chemical characteristics of this cofactor and reactions PLP potentially being able to mediate. Charge interactions, hydrogen bond formation, etc. play some essential roles in PLP-mediated reactions. In given buffer systems, there are plenty of these interactions, but many reactions, which PLP could catalyze when it is associated with protein component, do not proceed or are too slow to be apparent in any buffer system. When PLP is associated with proteins, many interacting factors, readily available in buffer systems, are eliminated. This likely magnifies tremendously the specific interactions of residues with a particular chemical group of PLP moiety and the substrate, thereby promoting specific reactions. Based on the important roles of these interactions, the structure-functional relationship and the mechanism study of PLP-dependent enzymes have become attractive fields of research in recent years. The research advances in several groups of PLP-dependent enzymes have been summarized in this review, with an emphasis on the residues and structural factors that are critical for catalysis and reaction specificity.

A better understanding of structure-function relationships of PLP-dependent enzymes has been achieved as indicated by this review, but there are still many questions left for being explored, e.g., the detailed O_2_ activation and attack mechanism remains unknown (Liang et al., 2017; Huang et al., 2018), whether the role of carbanionic intermediate in O_2_ activation (Huang et al., 2018) is a common mechanism shared in other PLP-involved oxidative reactions (Kaminaga et al., 2006; Torrens-Spence et al., 2012, 2013, 2014), the detailed steps of the product complex disintegration (Liang et al., 2017), and the functional characterization of some uncharacterized PLP-dependent enzymes (Vavricka et al., 2011; Liang et al., 2017).

## Author Contributions

JL wrote the manuscript. QH, YT, HD, and JYL contributed to the modification of the manuscript.

### Conflict of Interest Statement

The authors declare that the research was conducted in the absence of any commercial or financial relationships that could be construed as a potential conflict of interest.

## References

[B1] Beattie A. E. Clarke D. J. Wadsworth J. M. Lowther J. Sin H. L. Campopiano D. J. (2013). Reconstitution of the pyridoxal 5′-phosphate (PLP) dependent enzyme serine palmitoyltransferase (SPT) with pyridoxal reveals a crucial role for the phosphate during catalysis. Chem. Commun. 49, 7058–7060. 10.1039/c3cc43001d 23814788

[B2] Bell S. C. Turner J. M. (1977). Bacterial catabolism of threonine. Threonine degradation initiated by l-threonine acetaldehyde-lyase (aldolase) in species of Pseudomonas. Biochem. J. 166, 209–216. 911318 10.1042/bj1660209 PMC1164997

[B3] Berkovitch F. Behshad E. Tang K. H. Enns E. A. Frey P. A. Drennan C. L. (2004). A locking mechanism preventing radical damage in the absence of substrate, as revealed by the x-ray structure of lysine 5,6-aminomutase. Proc. Natl. Acad. Sci. U.S.A. 101, 15870–15875. 10.1073/pnas.0407074101 15514022 PMC528771

[B4] Burkhard P. Dominici P. Borri-Voltattorni C. Jansonius J. N. N. M.V. (2001). Structural insight into Parkinson's disease treatment from drug-inhibited DOPA decarboxylase. Nat. Struc. Biol. 8, 963–967. 10.1038/nsb1101-963 11685243

[B5] Canu N. Ciotti M. T. Pollegioni L. (2014). Serine racemase: a key player in apoptosis and necrosis. Front. Synaptic Neurosci. 6:9. 10.3389/fnsyn.2014.00009 24795622 PMC4000995

[B6] Cellini B. Montioli R. Oppici E. Astegno A. Voltattorni C. B. (2014). The chaperone role of the pyridoxal 5'-phosphate and its implications for rare diseases involving B6-dependent enzymes. Clin. Biochem. 47, 158–165. 10.1016/j.clinbiochem.2013.11.021 24355692

[B7] Chang Y. C. McCalmont T. Craves D. J. (1983). Functions of the 5'-phosphoryl group of pyridoxal 5'-phosphate in phosphorylase: a study using pyridoxal-reconstituted enzyme as a model system. Biochemistry 22, 4987–4993. 6639940 10.1021/bi00290a017

[B8] Chan-Huot M. Dos A. Zander R. Sharif S. Tolstoy P. M. Compton S.. (2013). NMR studies of protonation and hydrogen bond states of internal aldimines of pyridoxal 5'-phosphate acid-base in alanine racemase, aspartate aminotransferase, and poly-L-lysine. J. Am. Chem. Soc. 135, 18160–18175. 10.1021/ja408988z 24147985

[B9] Cho H. Ramaswamy S. Plapp B. V. (1997). Flexibility of liver alcohol dehydrogenase in stereoselective binding of 3-butylthiolane 1-oxides. Biochemistry 36, 382–389. 9003191 10.1021/bi9624604

[B10] Chow M. A. McElroy K. E. Corbett K. D. Berger J. M. Kirsch J. F. (2004). Narrowing substrate specificity in a directly evolved enzyme: the A293D mutant of aspartate aminotransferase. Biochemistry 43, 12780–12787. 10.1021/bi0487544 15461450

[B11] Christenson J. G. Dairman W. Udenfriend S. (1970). Preparation and properties of a homogeneous aromatic L-amino acid decarboxylase from hog kidney. Arch. Biochem. Biophys. 141, 356–367. 4991409 10.1016/0003-9861(70)90144-x

[B12] Clausen T. Huber R. Laber B. Pohlenz H.-D. Messerschmidt A. (1996). Crystal Structure of the Pyridoxal-5'-phosphate Dependent Cystathionine beta-lyase from *Escherichia coli* at 1.83 Å. J. Mol. Biol. 262, 202–224. 8831789 10.1006/jmbi.1996.0508

[B13] Cohen P. Duewer T. Fischer E. H. (1971). Phosphorylase from dogfish skeletal muscle. Purification and a comparison of its physical properties to those of rabbit muscle phosphorylase. Biochemistry 10, 2683–2694. 5558692 10.1021/bi00790a005

[B14] Connil N. Le Breton Y. Dousset X. Auffray Y. Rincé A. Prévost H. (2002). Identification of the *Enterococcus faecalis* tyrosine decarboxylase operon involved in tyramine production. Appl. Environ. Microbiol. 68, 3537–3544. 10.1128/AEM.68.7.3537-3544.2002 12089039 PMC126796

[B15] Contestabile R. Paiardini A. Pascarella S. di Salvo M. L. D'Aguanno S. Bossa F. (2001). l-Threonine aldolase, serine hydroxymethyltransferase and fungal alanine racemase. Eur. J. Biochem. 268, 6508–6525. 10.1046/j.0014-2956.2001.02606.x 11737206

[B16] Conti P. Tamborini L. Pinto A. Blondel A. Minoprio P. Mozzarelli A.. (2011). Drug discovery targeting amino acid racemases. Chem. Rev. 111, 6919–6946. 10.1021/cr2000702 21913633

[B17] Dajnowicz S. Johnston R. C. Parks J. M. Blakeley M. P. Keen D. A. Weiss K. L.. (2017). Direct visualization of critical hydrogen atoms in a pyridoxal 5'-phosphate enzyme. Nat. Commun. 8:955. 10.1038/s41467-017-01060-y 29038582 PMC5643538

[B18] Dean S. M. Greenberg W. A. Wong C.-H. (2007). Recent advances in aldolase-catalyzed asymmetric synthesis. Adv. Synth. Catalysis 349, 1308–1320. 10.1002/adsc.200700115

[B19] Devi S. Abdul Rehman S. A. Tarique K. F. Gourinath S. (2017). Structural characterization and functional analysis of cystathionine β-synthase: an enzyme involved in the reverse transsulfuration pathway of Bacillus anthracis. FEBS J. 284, 3862–3880. 10.1111/febs.14273 28921884

[B20] Di Salvo M. L. Florio R. Paiardini A. Vivoli M. D'Aguanno S. Contestabile R. (2013). Alanine racemase from *Tolypocladium inflatum*: a key PLP-dependent enzyme in cyclosporin biosynthesis and a model of catalytic promiscuity. Arch. Biochem. Biophys. 529, 55–65. 10.1016/j.abb.2012.11.011 23219598

[B21] Di Salvo M. L. Remesh S. G. Vivoli M. Ghatge M. S. Paiardini A. D'Aguanno S.. (2014). On the catalytic mechanism and stereospecificity of *Escherichia coli* l-threonine aldolase. FEBS J. 281, 129–145. 10.1111/febs.12581 24165453 PMC4366684

[B22] Duff S. M. Rydel T. J. McClerren A. L. Zhang W. Li J. Y. Sturman E. J.. (2012). The enzymology of alanine aminotransferase (AlaAT) isoforms from Hordeum vulgare and other organisms, and the HvAlaAT crystal structure. Arch. Biochem. Biophys. 528, 90–101. 10.1016/j.abb.2012.06.006 22750542

[B23] Dunathan H. C. (1966). Conformation and reaction specificity in pyridoxal phosphate enzymes. Proc. Natl. Acad. Sci. U.S.A. 55, 712–716. 5219675 10.1073/pnas.55.4.712 PMC224217

[B24] Eliot A. C. Kirsch J. F. (2004). Pyridoxal phosphate enzymes: mechanistic, structural, and evolutionary considerations. Annu. Rev. Biochem. 73, 383–415. 10.1146/annurev.biochem.73.011303.074021 15189147

[B25] Facchini P. J. De Luca V. (1995). Phloem-specific expression of tyrosine/dopa decarboxylase genes and the biosynthesis of isoquinoline alkaloids in opium poppy. Plant Cell 7, 1811–1821. 12242361 10.1105/tpc.7.11.1811 PMC161040

[B26] Facchini P. J. Huber-Allanach K. L. Tari L. W. (2000). Plant aromatic L-amino acid decarboxylases: evolution, biochemistry, regulation, and metabolic engineering applications. Phytochemistry 54, 121–138. 10.1002/chin.200036294 10872203

[B27] Falcicchio P. Wolterink-Van Loo S. Franssen M. C. van der Oost J. (2014). DHAP-dependent aldolases from (hyper)thermophiles: biochemistry and applications. Extremophiles 18, 1–13. 10.1007/s00792-013-0593-x 24166576

[B28] Fesko K. (2016). Threonine aldolases: perspectives in engineering and screening the enzymes with enhanced substrate and stereo specificities. Appl. Microbiol. Biotechnol. 100, 2579–2590. 10.1007/s00253-015-7218-5 26810201 PMC4761611

[B29] Fesko K. Strohmeier G. A. Breinbauer R. (2015). Expanding the threonine aldolase toolbox for the asymmetric synthesis of tertiary α-amino acids. Appl. Microbiol. Biotechnol. 99, 9651–9661. 10.1007/s00253-015-6803-y 26189018

[B30] Fesko K. Uhl M. Steinreiber J. Gruber K. Griengl H. (2009). Biocatalytic Access to α,α-Dialkyl-α-amino acids by a mechanism-based approach. Angewandte Chemie Int. Edn. 49, 121–124. 10.1002/anie.200904395 19943295

[B31] Fogle E. J. Toney M. D. (2010). Mutational analysis of substrate interactions with the active site of dialkylglycine decarboxylase. Biochemistry 49, 6485–6493. 10.1021/bi100648w 20540501 PMC2994807

[B32] Frey P. A. (2001). Radicalmechanisms of enzymatic catalysis. Annu. Rev. Biochem. 70, 121–148. 10.1146/annurev.biochem.70.1.121 11395404

[B33] Fujioka M. (1969). Purification and properties of serine hydroxymethylase from soluble and mitochondrial fractions of rabbit liver. Biochim. Biophys. Acta 185, 338–349. 10.1016/0005-2744(69)90427-6 5808700

[B34] Fukui T. Soda K. (2008). Molecular Aspects of Enzyme Catalysis. Tokyo: Wiley-Blackwell, 186–190.

[B35] Giardina G. Montioli R. Gianni S. Cellini B. Paiardini A. Voltattorni C. B.. (2011). Open conformation of human DOPA decarboxylase reveals the mechanism of PLP addition to Group II decarboxylases. Proc. Natl. Acad. Sci. U.S.A. 108, 20514–20519. 10.1073/pnas.1111456108 22143761 PMC3251144

[B36] Goldberg J. M. Kirsch J. F. (1996). The reaction catalyzed by *Escherichia coli* aspartate aminotransferase has multiple partially rate-determining steps, while that catalyzed by the Y225F mutant is dominated by ketimine hydrolysis. Biochemistry 35, 5280–5291. 8611515 10.1021/bi952138d

[B37] Goto M. Yamauchi T. Kamiya N. Miyahara I. Yoshimura T. Mihara H.. (2009). Crystal structure of a homolog of mammalian serine racemase from Schizosaccharomyces pombe. J. Biol. Chem. 284, 25944–25952. 10.1074/jbc.M109.010470 19640845 PMC2757995

[B38] Grishin N. V. Phillips M. A. Goldsmith E. J. (1995). Modeling of the spatial structure of eukaryotic ornithine decarboxylases. Protein Sci. 4, 1291–1304. 10.1002/pro.5560040705 7670372 PMC2143167

[B39] Gupta S. Kelow S. Wang L. Andrake M. Dunbrack R. L. Jr. Kruger W. D. (2018). Mouse modeling and structural analysis of the p.G307S mutation in human cystathionine beta-synthase (CBS) reveal effects on CBS activity but not stability. J. Biol. Chem. 293, 13921–13931. 10.1074/jbc.RA118.002164 30030379 PMC6130948

[B40] Han L. Schwabacher A. W. Moran G. R. Silvaggi N. R. (2015). Streptomyces wadayamensis MppP is a Pyridoxal 5'-Phosphate-Dependent L-Arginine alpha-Deaminase, gamma-Hydroxylase in the Enduracididine Biosynthetic Pathway. Biochemistry 54, 7029–7040. 10.1021/acs.biochem.5b01016 26551990

[B41] Han Q. Cai T. Tagle D. A. Li J. (2010a). Structure, expression, and function of kynurenine aminotransferases in human and rodent brains. Cell Mol. Life Sci. 67, 353–368. 10.1007/s00018-009-0166-4 19826765 PMC2867614

[B42] Han Q. Cai T. Tagle D. A. Robinson H. Li J. (2008a). Substrate specificity and structure of human aminoadipate aminotransferase/kynurenine aminotransferase II. Biosci. Rep. 28, 205–215. 10.1042/BSR20080085 18620547 PMC2559858

[B43] Han Q. Ding H. Robinson H. Christensen B. M. Li J. (2010b). Crystal structure and substrate specificity of *Drosophila* 3,4-dihydroxyphenylalanine decarboxylase. PLoS ONE 5:e8826. 10.1371/journal.pone.0008826 20098687 PMC2809104

[B44] Han Q. Robinson H. Li J. (2008b). Crystal structure of human kynurenine aminotransferase II. J. Biol. Chem. 283, 3567–3573. 10.1074/jbc.M708358200 18056995

[B45] Hayashi H. Mizuguchi H. Kagamiyama H. (1993). Rat liver aromatic L-amino acid decarboxylase: spectroscopic and kinetic analysis of the coenzyme and reaction intermediates. Biochemistry 32, 812–818. 10.1021/bi00054a011 8422386

[B46] Helmreich E. J. (1992). How pyridoxal 5'-phosphate could function in glycogen phosphorylase catalysis. Biofactors 3, 159–172. 1599610

[B47] Hernandez K. Zelen I. Petrillo G. Usón I. Wandtke C. M. Bujons J.. (2015). Engineered L-Serine Hydroxymethyltransferase from *Streptococcus thermophilus* for the synthesis of α,α-Dialkyl-α-Amino Acids. Angewandte Chem. Int. Edn. 54, 3013–3017. 10.1002/anie.201411484 25611820

[B48] Huang Y. Liu X. Cui Z. Wiegmann D. Niro G. Ducho C.. (2018). Pyridoxal-5'-phosphate as an oxygenase cofactor: discovery of a carboxamide-forming, alpha-amino acid monooxygenase-decarboxylase. Proc. Natl. Acad. Sci. U.S.A. 115, 974–979. 10.1073/pnas.1718667115 29343643 PMC5798378

[B49] Hullo M.-F. Auger S. Soutourina O. Barzu O. Yvon M. Danchin A.. (2007). Conversion of methionine to cysteine in *Bacillus subtilis* and its regulation. J. Bacteriol. 189, 187–189. 10.1128/JB.01273-06 17056751 PMC1797209

[B50] Jebai F. Hanoun N. Hamon M. Thibault J. Peltre G. Gros F.. (1997). Expression, purification, and characterization of rat aromaticl-amino acid decarboxylase in *Escherichia coli*. Protein Exp. Purification 11, 185–194. 9367815 10.1006/prep.1997.0778

[B51] Jhee K.-H. Yang L.-H. Ahmed S. A. McPhie P. Rowlett R. Miles E. W. (1998). Mutation of an active site residue of tryptophan synthase (β-Serine 377) alters cofactor chemistry. J. Biol. Chem. 273, 11417–11422. 10.1074/jbc.273.19.11417 9565551

[B52] Käck H. Sandmark J. Gibson K. Schneider G. Lindqvist Y. (1999). Crystal structure of diaminopelargonic acid synthase: evolutionary relationships between pyridoxal-5'-phosphate-dependent enzymes. J. Mol. Biol. 291, 857–876. 10452893 10.1006/jmbi.1999.2997

[B53] Kaminaga Y. Schnepp J. Peel G. Kish C. M. Ben-Nissan G. Weiss D.. (2006). Plant phenylacetaldehyde synthase is a bifunctional homotetrameric enzyme that catalyzes phenylalanine decarboxylation and oxidation. J. Biol. Chem. 281, 23357–23366. 10.1074/jbc.M602708200 16766535

[B54] Kappes B. Tews I. Binter A. Macheroux P. (2011). PLP-dependent enzymes as potential drug targets for protozoan diseases. Biochim. Biophys. Acta 1814, 1567–1576. 10.1016/j.bbapap.2011.07.018 21884827

[B55] Kern A. D. Oliveira M. A. Coffino P. Hackert M. L. (1999). Structure of mammalian ornithine decarboxylase at 1.6 A resolution: stereochemical implications of PLP-dependent amino acid decarboxylases. Structure 7, 567–581. 10378276 10.1016/s0969-2126(99)80073-2

[B56] Kielkopf C. L. Burley S. K. (2002). X-ray structures of threonine aldolase complexes: structural basis of substrate recognition. Biochemistry 41, 11711–11720. 10.1021/bi020393+ 12269813

[B57] Komori H. Nitta Y. Ueno H. Higuchi Y. (2012). Structural study reveals that Ser-354 determines substrate specificity on human histidine decarboxylase. J. Biol. Chem. 287, 29175–29183. 10.1074/jbc.M112.381897 22767596 PMC3436558

[B58] Koutmos M. Kabil O. Smith J. L. Banerjee R. (2010). Structural basis for substrate activation and regulation by cystathionine beta-synthase (CBS) domains in cystathionine {beta}-synthase. Proc. Natl. Acad. Sci. U.S.A. 107, 20958–20963. 10.1073/pnas.1011448107 21081698 PMC3000283

[B59] Koyanagi T. Nakagawa A. Sakurama H. Yamamoto K. Sakurai N. Takagi Y.. (2012). Eukaryotic-type aromatic amino acid decarboxylase from the root colonizer *Pseudomonas putida* is highly specific for 3,4-dihydroxyphenyl-L-alanine, an allelochemical in the rhizosphere. Microbiology 158(Pt 12), 2965–2974. 10.1099/mic.0.062463-0 23059975

[B60] Larsson S. C. Orsini N. Wolk A. (2010). Vitamin B6 and risk of colorectal cancer: a meta-analysis of prospective studies. J. Am. Med. Assoc. 303, 1077–1083. 10.1001/jama.2010.263 20233826

[B61] Lepore B. W. Ruzicka F. J. Frey P. A. Ringe D. (2005). The x-ray crystal structure of lysine-2,3-aminomutase from *Clostridium subterminale*. Proc. Natl. Acad. Sci. U.S.A. 102, 13819–13824. 10.1073/pnas.0505726102 16166264 PMC1236562

[B62] Liang J. Han Q. Ding H. Li J. (2017). Biochemical identification of residues that discriminate between 3,4-dihydroxyphenylalanine decarboxylase and 3,4-dihydroxyphenylacetaldehyde synthase-mediated reactions. Insect. Biochem. Mol. Biol. 91, 34–43. 10.1016/j.ibmb.2017.10.001 29037755

[B63] Livanova N. B. Chebotareva N. A. Eronina T. B. Kurganov B. I. (2002). Pyridoxal 5'-phosphate as a catalytic and conformational cofactor of muscle glycogen phosphorylase. Biochemistry 67, 1089–1098. 10.1023/A:1020978825802 12460107

[B64] Major D. T. Gao J. (2006). A combined quantum mechanical and molecular mechanical study of the reaction mechanism and α-amino acidity in alanine racemase. J. Am. Chem. Soc. 128, 16345–16357. 10.1021/ja066334r 17165790

[B65] Manta B. Cassimjee K. E. Himo F. (2017). Quantum chemical study of dual-substrate recognition in omega-transaminase. ACS Omega 2, 890–898. 10.1021/acsomega.6b00376 30023618 PMC6044752

[B66] Matoba Y. Yoshida T. Izuhara-Kihara H. Noda M. Sugiyama M. (2017). Crystallographic and mutational analyses of cystathionine beta-synthase in the H2 S-synthetic gene cluster in *Lactobacillus plantarum*. Protein Sci. 26, 763–783. 10.1002/pro.3123 28127810 PMC5368070

[B67] Mehta P. K. Hale T. I. Christen P. (1993). Aminotransferases: demonstration of homology and division into evolutionary subgroups. Eur. J. Biochem. 214, 549–561. 8513804 10.1111/j.1432-1033.1993.tb17953.x

[B68] Milano T. Paiardini A. Grgurina I. Pascarella S. (2013). Type I pyridoxal 5′-phosphate dependent enzymatic domains embedded within multimodular nonribosomal peptide synthetase and polyketide synthase assembly lines. BMC Struc. Biol. 13:26. 10.1186/1472-6807-13-26 24148833 PMC3870968

[B69] Miles E. W. Kraus J. P. (2004). Cystathionine beta-synthase: structure, function, regulation, and location of homocystinuria-causing mutations. J. Biol. Chem. 279, 29871–29874. 10.1074/jbc.R400005200 15087459

[B70] Milić D. Demidkina T. V. Zakomirdina L. N. Matković-Calogović D. Antson A. A. (2012). Crystal structure of *Citrobacter freundii* Asp214Ala tyrosine phenollyase reveals that Asp214 is critical for maintaining a strain in the internal aldimine. Croatica Chem. Acta 85, 283–288. 10.5562/cca1915

[B71] Moreno-Arribas V. Lonvaud-Funel A. (2001). Purification and characterization of tyrosine decarboxylase of *Lactobacillus brevis* IOEB 9809 isolated from wine. FEMS Microbiol. Lett. 195, 103–107. 10.1016/S0378-1097(00)00559-0 11167003

[B72] Nasir N. Anant A. Vyas R. Biswal B. K. (2016). Crystal structures of *Mycobacterium tuberculosis* HspAT and ArAT reveal structural basis of their distinct substrate specificities. Sci. Rep. 6:18880. 10.1038/srep18880 26738801 PMC4703992

[B73] Nelson D. L. Applegate G. A. Beio M. L. Graham D. L. Berkowitz D. B. (2017). Human serine racemase structure/activity relationship studies provide mechanistic insight and point to position 84 as a hot spot for beta-elimination function. J. Biol. Chem. 292, 13986–14002. 10.1074/jbc.M117.777904 28696262 PMC5572919

[B74] Ngo H. P. Cerqueira N. M. Kim J. K. Hong M. K. Fernandes P. A. Ramos M. J.. (2014). PLP undergoes conformational changes during the course of an enzymatic reaction. Acta Crystallogr. D Biol. Crystallogr. 70(Pt 2), 596–606. 10.1107/S1399004713031283 24531493

[B75] Nitoker N. Major D. T. (2015). Understanding the reaction mechanism and intermediate stabilization in mammalian serine racemase using multiscale quantum-classical simulations. Biochemistry 54, 516–527. 10.1021/bi500984m 25493718

[B76] Niu W. Wang J. Qian J. Wang M. Wu P. Chen F.. (2018). Allosteric control of human cystathionine beta-synthase activity by a redox active disulfide bond. J. Biol. Chem. 293, 2523–2533. 10.1074/jbc.RA117.000103 29298893 PMC5818181

[B77] Okada K. Hirotsu K. Sato M. Hayashi H. Kagamiyama H. (1997). Three-dimensional structure of *Escherichia coli* branched-chain amino acid aminotransferase at 2.5 A resolution. J. Biochem. 121, 637–641. 9163511 10.1093/oxfordjournals.jbchem.a021633

[B78] Ondrechen M. J. Briggs J. M. McCammon J. A. (2001). A model for enzyme–substrate interaction in alanine racemase. J. Am. Chem. Soc. 123, 2830–2834. 10.1021/ja0029679 11456969

[B79] Park E. S. Kim M. Shin J. S. (2012). Molecular determinants for substrate selectivity of omega-transaminases. Appl. Microbiol. Biotechnol. 93, 2425–2435. 10.1007/s00253-011-3584-9 21983703

[B80] Parrish R. F. Uhing R. J. Graves D. J. (1977). Effect of phosphate analogues on the activity of pyridoxal reconstituted glycogen phosphorylase. Biochemistry 16, 4824–4831. 911792 10.1021/bi00641a011

[B81] Percudani R. Peracchi A. (2009). The B6 database: a tool for the description and classification of vitamin B6-dependent enzymatic activities and of the corresponding protein families. BMC Bioinform. 10:273. 10.1186/1471-2105-10-273. 19723314 PMC2748086

[B82] Phillips R. S. Scott I. Paulose R. Patel A. Barron T. C. (2014). The phosphate of pyridoxal-5'-phosphate is an acid/base catalyst in the mechanism of *Pseudomonas fluorescens* kynureninase. FEBS J. 281, 1100–1109. 10.1111/febs.12671 24304904

[B83] Qin H.-M. Imai F. L. Miyakawa T. Kataoka M. Kitamura N. Urano N.. (2014). l-allo-Threonine aldolase with an H128Y/S292R mutation from *Aeromonas jandaei* DK-39 reveals the structural basis of changes in substrate stereoselectivity. Acta Crystallogr. D 70, 1695–1703. 10.1107/S1399004714007664 24914980

[B84] Rausch C. Lerchner A. Schiefner A. Skerra A. (2012). Crystal structure of the ω-aminotransferase from *Paracoccus denitrificans* and its phylogenetic relationship with other class III amino- transferases that have biotechnological potential. Proteins 81, 774–787. 10.1002/prot.24233 23239223

[B85] Ringel M. T. Drager G. Bruser T. (2016). PvdN enzyme catalyzes a periplasmic pyoverdine modification. J. Biol. Chem. 291, 23929–23938. 10.1074/jbc.M116.755611 27703013 PMC5104919

[B86] Rubí B. (2012). Pyridoxal 5'-phosphate (PLP) deficiency might contribute to the onset of type I diabetes. Med. Hypotheses 78, 179–182. 10.1016/j.mehy.2011.10.021 22088923

[B87] Rubinstein A. Major D. T. (2010). Understanding catalytic specificity in alanine racemase from quantum mechanical and molecular mechanical simulations of the arginine 219 Mutant. Biochemistry 49, 3957–3964. 10.1021/bi1002629 20394349

[B88] Schneider G. Käck H. Lindqvist Y. (2000). The manifold of vitamin B6 dependent enzymes. Structure 8, R1–R6. 10.1016/s0969-2126(00)00085-x 10673430

[B89] Seebeck F. P. Hilvert D. (2003). Conversion of a PLP-dependent racemase into an aldolase by a single active site mutation. J. Am. Chem. Soc. 125, 10158–10159. 10.1021/ja036707d 12926923

[B90] Shostak K. Schirch V. (1988). Serine hydroxymethyltransferase: mechanism of the racemization and transamination of D- and L-alanine. Biochemistry 27, 8007–8014. 10.1021/bi00421a006 3069126

[B91] Singh S. Padovani D. Leslie R. A. Chiku T. Banerjee R. (2009). Relative contributions of cystathionine beta-synthase and gamma-cystathionase to H2S biogenesis via alternative trans-sulfuration reactions. J. Biol. Chem. 284, 22457–22466. 10.1074/jbc.M109.010868 19531479 PMC2755967

[B92] Sivaraman J. Li Y. Larocque R. Schrag J. D. Cygler M. Matte A. (2001). Crystal structure of histidinol phosphate aminotransferase (HisC) from *Escherichia coli*, and its covalent complex with pyridoxal-5'-phosphate and l-histidinol phosphate. J. Mol. Biol. 311, 761–776. 10.1006/jmbi.2001.4882 11518529

[B93] Smith M. A. Mack V. Ebneth A. Moraes I. Felicetti B. Wood M.. (2010). The structure of mammalian serine racemase: evidence for conformational changes upon inhibitor binding. J. Biol. Chem. 285, 12873–12881. 10.1074/jbc.M109.050062 20106978 PMC2857111

[B94] Spies M. A. Toney M. D. (2003). Multiple hydrogen kinetic isotope effects for enzymes catalyzing exchange with solvent: application to alanine racemase. Biochemistry 42, 5099–5107. 10.1021/bi0274064 12718553

[B95] Spies M. A. Woodward J. J. Watnik M. R. Toney M. D. (2004). Alanine racemase free energy profiles from global analyses of progress curves. J. Am. Chem. Soc. 126, 7464–7475. 10.1021/ja049579h 15198593

[B96] Steffen-Munsberg F. Vickers C. Kohls H. Land H. Mallin H. Nobili A.. (2015). Bioinformatic analysis of a PLP-dependent enzyme superfamily suitable for biocatalytic applications. Biotechnol. Adv. 33, 566–604. 10.1016/j.biotechadv.2014.12.012 25575689

[B97] Strych U. Davlieva M. Longtin J. P. Murphy E. L. Im H. Benedik M. J.. (2007). Purification and preliminary crystallization of alanine racemase from *Streptococcus pneumoniae*. BMC Microbiol. 7:40. 10.1186/1471-2180-7-40 17509154 PMC1885262

[B98] Sun X. Li H. Alfermann J. Mootz H. D. Yang H. (2014). Kinetics profiling of gramicidin S synthetase A, a member of nonribosomal peptide synthetases. Biochemistry 53, 7983–7989. 10.1021/bi501156m 25437123

[B99] Taylor J. L. Price J. E. Toney M. D. (2015). Directed evolution of the substrate specificity of dialkylglycine decarboxylase. Biochim. Biophys. Acta 1854, 146–155. 10.1016/j.bbapap.2014.12.003 25500286 PMC4334570

[B100] Toney M. D. (2011). Controlling reaction specificity in pyridoxal phosphate enzymes. Biochim. Biophys. Acta 1814, 1407–1418. 10.1016/j.bbapap.2011.05.019 21664990 PMC3359020

[B101] Toney M. D. (2014). Aspartate aminotransferase: an old dog teaches new tricks. Arch. Biochem. Biophys. 544, 119–127. 10.1016/j.abb.2013.10.002 24121043 PMC3946379

[B102] Toney M. D. Kirsch J. F. (1993). Lysine 258 in aspartate aminotransferase: enforcer of the Circe effect for amino acid substrates and the general-base catalyst for the 1,3-prototropic shift. Biochemistry 32, 1471–1479. 10.1021/bi00057a010 8431426

[B103] Torrens-Spence M. P. Gillaspy G. Zhao B. Harich K. White R. H. Li J. (2012). Biochemical evaluation of a parsley tyrosine decarboxylase results in a novel 4-hydroxyphenylacetaldehyde synthase enzyme. Biochem. Biophys. Res. Commun. 418, 211–216. 10.1016/j.bbrc.2011.12.124 22266321

[B104] Torrens-Spence M. P. Liu P. Ding H. Harich K. Gillaspy G. Li J. (2013). Biochemical evaluation of the decarboxylation and decarboxylation-deamination activities of plant aromatic amino acid decarboxylases. J. Biol. Chem. 288, 2376–2387. 10.1074/jbc.M112.401752 23204519 PMC3554908

[B105] Torrens-Spence M. P. von Guggenberg R. Lazear M. Ding H. Li J. (2014). Diverse functional evolution of serine decarboxylases: identification of two novel acetaldehyde synthases that uses hydrophobic amino acids as substrates. BMC Plant Biol. 14:247. 10.1186/s12870-014-0247-x 25230835 PMC4177580

[B106] Uhl M. K. Oberdorfer G. Steinkellner G. Riegler-Berket L. Mink D. van Assema F.. (2015). The crystal structure of D-threonine aldolase from alcaligenes xylosoxidans provides insight into a metal ion assisted PLP-dependent mechanism. PLoS ONE 10:e0124056. 10.1371/journal.pone.0124056 25884707 PMC4401734

[B107] Vavricka C. Han Q. Huang Y. Erickson S. M. Harich K. Christensen B. M.. (2011). From L-dopa to dihydroxyphenylacetaldehyde: a toxic biochemical pathway plays a vital physiological function in insects. PLoS ONE 6:e16124. 10.1371/journal.pone.0016124 21283636 PMC3026038

[B108] Wang Z. X. (1999). Kinetic study on the dimer-tetramer interconvertion of glycogen phosphorylase. Eur. J. Biochem. 259, 609–617. 10092844 10.1046/j.1432-1327.1999.00058.x

[B109] Watanabe A. Yoshimura T. Mikami B. Esaki N. (1999). Tyrosine 265 of alanine racemase serves as a base abstracting &alpha;-hydrogen from L-alanine: the counterpart residue to Lysine 39 Specific to D-Alanine. J. Biochem. 126, 781–786. 10502689 10.1093/oxfordjournals.jbchem.a022517

[B110] Watanabe A. Yoshimura T. Mikami B. Hayashi H. Kagamiyama H. Esaki N. (2002). Reaction mechanism of alanine racemase from *Bacillus stearothermophilus*: x-ray crystallographic studies of the enzyme bound with N-(5'-phosphopyridoxyl)alanine. J. Biol. Chem. 277, 19166–19172. 10.1074/jbc.M201615200 11886871

[B111] Williams B. B. Van Benschoten A. H. Cimermancic P. Donia M. S. Zimmermann M. Taketani M.. (2014). Discovery and characterization of gut microbiota decarboxylases that can produce the neurotransmitter tryptamine. Cell Host Microbe 16, 495–503. 10.1016/j.chom.2014.09.001 25263219 PMC4260654

[B112] Yoshimura T. Goto M. (2008). d-Amino acids in the brain: structure and function of pyridoxal phosphate-dependent amino acid racemases. FEBS J. 275, 3527–3537. 10.1111/j.1742-4658.2008.06516.x 18564179

[B113] Zhu H. Xu G. Zhang K. Kong X. Han R. Zhou J.. (2016). Crystal structure of tyrosine decarboxylase and identification of key residues involved in conformational swing and substrate binding. Sci. Rep. 6:27779. 10.1038/srep27779 27292129 PMC4904194

